# Distinct and Cooperative Functions for the *Protocadherin*-α, -β and -γ Clusters in Neuronal Survival and Axon Targeting

**DOI:** 10.3389/fnmol.2016.00155

**Published:** 2016-12-23

**Authors:** Sonoko Hasegawa, Makiko Kumagai, Mitsue Hagihara, Hiroshi Nishimaru, Keizo Hirano, Ryosuke Kaneko, Atsushi Okayama, Teruyoshi Hirayama, Makoto Sanbo, Masumi Hirabayashi, Masahiko Watanabe, Takahiro Hirabayashi, Takeshi Yagi

**Affiliations:** ^1^KOKORO-Biology Group, Laboratories for Integrated Biology, Graduate School of Frontier Biosciences, Osaka UniversitySuita, Japan; ^2^AMED-CREST, Japan Agency for Medical Research and Development (AMED)Suita, Japan; ^3^Division of Biomedical Science, Faculty of Medicine, University of TsukubaTsukuba, Japan; ^4^Bioresource Center, Graduate School of Medicine, Gunma UniversityMaebashi, Japan; ^5^Section of Mammalian Transgenesis, Center for Genetic Analysis of Behavior, National Institute for Physiological SciencesOkazaki, Japan; ^6^Department of Anatomy, Hokkaido University Graduate School of MedicineSapporo, Japan

**Keywords:** protocadherin, olfactory axon, apoptosis, gene targeting, brainstem, spinal cord, cell adhesion molecule, locomotion

## Abstract

The clustered *protocadherin* (*Pcdh*) genes are divided into the *Pcdh*α*, Pcdh*β, and *Pcdh*γ clusters. Gene-disruption analyses in mice have revealed the *in vivo* functions of the *Pcdh*α and *Pcdh*γ clusters. However, all Pcdh protein isoforms form combinatorial *cis*-hetero dimers and enter *trans*-homophilic interactions. Here we addressed distinct and cooperative functions in the *Pcdh* clusters by generating six cluster-deletion mutants (Δα, Δβ, Δγ, Δ*αβ*, Δ*βγ*, and Δ*αβγ*) and comparing their phenotypes: Δα, Δβ, and Δ*αβ* mutants were viable and fertile; Δγ mutants lived less than 12 h; and Δ*βγ* and Δ*αβγ* mutants died shortly after birth. The *Pcdh*α*, Pcdh*β, and *Pcdh*γ clusters were individually and cooperatively important in olfactory-axon targeting and spinal-cord neuron survival. Neurodegeneration was most severe in Δ*αβγ* mutants, indicating that *Pcdh*α and *Pcdh*β function cooperatively for neuronal survival. The *Pcdh*α*, Pcdh*β, and *Pcdh*γ clusters share roles in olfactory-axon targeting and neuronal survival, although to different degrees.

## Introduction

The clustered protocadherins (Pcdhs) are diverse cadherin-related receptors and constitute gene clusters in mammals (Kohmura et al., 1998; Wu and Maniatis, 1999). The *Pcdh* genes are expressed in the central and peripheral nervous systems, and their combinatorial and differential expression in individual neurons generates neuronal identity based on tremendous cell-surface diversity (Zipursky and Sanes, 2010; Weiner and Jontes, 2013; Yagi, 2014).

Mammals have over 50 genes located in the *Pcdh*α*, Pcdh*β, and *Pcdh*γ gene clusters, which are all on the same chromosome. In the genome structure, the *Pcdh*α or *Pcdh*γ clusters include constant exons that are commonly spliced to variable exons. Thus, all Pcdhα or Pcdhγ proteins have a common cytoplasmic tail; this does not in occur in the *Pcdh*β cluster. Both the Pcdhα and Pcdhγ cytoplasmic tails commonly interact with two tyrosine kinases, focal adhesion kinase (FAK) and PYK2, *in vitro* and *in vivo* (Chen et al., 2009). In contrast, Pcdhβ proteins do not have the common cytoplasmic tail and do not bind FAK or PYK2.

Mice have 58 *Pcdh* members—14 in *Pcdh*α, 22 in *Pcdh*β, and 22 in *Pcdh*γ. *Five* C-type isoforms, α*C1*, α*C2*, γ*C3*, γ*C4*, and γ*C5* are constitutively expressed in neurons; the remaining 53 members of *Pcdh*α*, Pcdh*β, and *Pcdh*γ are stochastically expressed in individual neurons (Esumi et al., 2005; Kaneko et al., 2006; Hirano et al., 2012). All of the isoform members engage in homophilic protein-protein interactions to induce cell adhesion and form distinctive combinations of *cis*-heteromeric dimers for *trans*-homophilic cell-surface interactions (Schreiner and Weiner, 2010; Thu et al., 2014; Nicoludis et al., 2015; Rubinstein et al., 2015; Goodman et al., 2016).

Studies of the individual *Pcdh* gene clusters using genetic approaches in mice have provided functional evidence that the clustered Pcdhs are required for normal development of the nervous system. Loss of the *Pcdh*α gene cluster's functions in mice induces defects in olfactory sensory-axon coalescence, serotonergic axon arborization, retinogeniculate axon targeting, and in the cortico-cortical pathway between the primary somatosensory cortices in both hemispheres, as well as impaired fear learning and sensory integrations of perceptual space, which occur in the cortex (Fukuda et al., 2008; Hasegawa et al., 2008, 2012; Katori et al., 2009; Yoshitake et al., 2013; Yamashita et al., 2012; Meguro et al., 2005). On the other hand, *Pcdh*γ mutants die after birth with repetitive tremors associated with massive interneuron apoptosis and synapse loss in the spinal cord (Wang et al., 2002; Weiner et al., 2005; Prasad et al., 2008). The Ia afferent terminal arborizations appear to be clumped around motor neurons in these mutants (Prasad and Weiner, 2011; Chen et al., 2012). *Pcdh*γ-deletion mutants are well-known to show dendritic arborization phenotypes. For example, in cortical and hippocampal neurons, *Pcdh*γ deletion causes simplified dendritic arbors (Garrett et al., 2012; Suo et al., 2012), and a similar but less severe phenotype is seen in *Pcdh*α-deficient hippocampal neurons *in vitro* and *in vivo* (Suo et al., 2012). Although these genetic studies provide interesting insights into the roles of the *Pcdh* clusters in the mouse brain, the distinct or cooperative functions of the diverse isoforms encoded by the *Pcdh*α*, Pcdh*β, and *Pcdh*γ gene clusters is not understood. Interestingly, no *in vivo* abnormalities have been reported for the *Pcdh*β cluster.

In the present study, we generated various combinations of deletion-mutant mice lacking entire *Pcdh* clusters and comprehensively analyzed clusters' distinct and cooperative functions *in vivo*. The *SyCP-Cre* driver, which is based on homologous chromosomal pairing during meiosis, has been used to generate *trans*-allelic recombinations in mice (Hérault et al., 1998; Noguchi et al., 2009). Using this method, we here produced variable-deletion mutants among the *Pcdh*α*, Pcdh*β, and *Pcdh*γ gene clusters. A *TAF7* gene located between the *Pcdh*β and *Pcdh*γ clusters in the genome is essential for early embryonic development (Gegonne et al., 2012); thus, to produce variable combinations of *Pcdh*-cluster deletion mutants, we rescued the TAF7 expression using its transgenic mouse. Finally, we obtained variable single, double, and triple *Pcdh*-cluster-deficient mutants. This study presents the distinct and cooperative functions among the *Pcdh* clusters in the formation of functional neuronal circuits.

## Materials and methods

### Animal experiments

All the experimental procedures were in accordance with the Guide for the Care and Use of Laboratory Animals of the Science Council of Japan and were approved by the Animal Experiment Committee of Osaka University.

### Generation of mutant mice with *loxP*-site insertions

All the primer sets mentioned below can be found in Supplementary Table S1. To insert *loxP* sites into the *Pcdh* gene cluster, we generated each targeting vector using the Red recombination system (Liu et al., 2003). BAC modifications were performed by transferring purified mouse BAC RP23-303I8 for the α*1MV* targeting vector, BAC RP23-318M13 for the α*CR-loxP* and β*1-loxP* targeting vectors, and BAC RP23-62D17 for the β*22-loxP* targeting vector into *E. coli* EL350 cells. To construct the α*1MV* targeting vector, we introduced *loxP* sites upstream of the *Pcdh*α cluster and a Myc-tagged Venus fluorescent protein gene into the *Pcdh*α*1* exon. The homologous fragments of the targeting sites were inserted into the pBTloxP2 plasmid, which contains a floxed *neo*^*r*^ cassette. Homology arms were generated by PCR-amplification of the mouse BAC. The *Acc*I fragment of the 5′ homology fragments was amplified using a1MVA-Fanda1MVA-R primers and was subcloned into the *Cla*I restriction site of pBTloxP2. The Myc-tagged Venus-fused 3′ homology fragment consisted of a *Cla*I-digested amplicon by the a1MVB-Fanda1MVB-R primers; *Cla*I, a *Sbf* I-digested amplicon by the a1MVBC-Fanda1MVC-R primers with Venus cDNA as a template, and the *Sbf* I-digested amplicon by the a1MVD-Fanda1MVBD-R primers. The Myc-tagged Venus-fused 3′ homology fragment was subcloned into the *Bam*HI and *Sac*I restriction sites of pBTloxP2. The floxed *neo*^*r*^ gene with the Myc-tagged Venus gene and the homology arms was excised by *Sal*I digestion and gel-purified. The purified *neo*^*r*^ cassette was electroporated into EL350 cells containing RP23-303I8 that had been induced for Red recombination functions by prior growth at 42°C for 15 min. Transformants were selected on plates containing Kanamycin. The modified BACs were verified by PCR. Finally, homologous recombination was used to insert BAC DNA fragments containing the Myc-tagged Venus and *neo*^*r*^ gene into pBRSDT, which contained the gene for diphtheria toxin A (DT-A) in the *Sal*I site of pBR322 (Yagi et al., 1990; Yanagawa et al., 1999). The homology fragments for this recombination were amplified by the a1MVE-F and a1MVE-R primers and the a1MVF-F and a1MVF-R primers, and were subcloned into pBRSDT at the *Hin*dIII and *Nhel* restriction sites. We retrieved the 16-kb BAC DNA fragments that contained the Myc-tagged Venus and floxed *neo*^*r*^ genes, and inserted them into pBRSDT. The retrieved plasmid was used as a targeting vector (Tarusawa et al., 2016).

A similar method was used to construct the α*CR-loxP*, β*1-loxP*, and β*22-loxP* targeting vectors. For the α*CR-loxP* targeting vector, the two homology arms subcloned into the pBTloxP plasmid were generated by PCR with the CP3/BT5-F2 and CP3/BT5-R primers and the CP3/BT3-F and CP3/BT3-R primers. To retrieve the targeting vector from the BAC clone that included the targeted *neo*^*r*^ gene, two homology arms were generated by PCR with the CP3/RT5C-F and CP3/RT5C-R primers and the CP3/RT3C-F and CP3/RT3C-R primers and subcloned into pBRSDT.

For the β*1-loxP* targeting vector, two homology arms were generated by PCR with the b1loxA-F and b1loxA-R primers and the b1loxB-F and b1loxB-R primers, and two homology arms were generated by PCR with the b1loxC-F and the b1loxC-R primers and the b1loxD-F and b1loxD-R primers. The homology arms were subcloned into pBTloxP2 and pBRSDT, respectively.

Similarly, for the β*22-loxP* targeting vector, two homology arms were generated by PCR with the b22loxA-F and b22loxA-R primers and the b22loxB-F and b22loxB-R primers, and two homology arms generated by PCR with the b22loxC-F and b22loxC-R primers and the b22loxD-F and b22loxD-R primers were subcloned into pBTloxP2 and pBRSDT, respectively.

Each linearized targeting vector was electroporated into TT2 ES cells, and cells carrying the mutant alleles were screened for recombination of ES cells by Southern hybridization with a probe isolated by PCR using mouse BAC as a template. Recombinant ES-cell clones were injected into ICR blastocysts, and the male chimeras were bred with C57BL/6 mice. The primer sequences used to construct the targeting vector and the isolation of the probes for Southern hybridization are shown in Supplementary Table S1. The generation of γ*LacZ* mutant mice, in which the *lacZ* gene and the *loxP* site were inserted downstream of the γ*CR3* exon, was described previously (Yokota et al., 2011).

### Generation of *Pcdh-*cluster deletion-mutant mice

The cluster-deletion alleles *Pcdha*^*del*^*, Pcdhb*^*del*^, *Pcdhg*^*del*^*, Pcdhab*^*del*^*, Pcdhbg*^*del*^, and *Pcdhabg*^*del*^ were generated by Cre-induced meiotic recombination between α*1MV*, β*1-loxP*, β*22-loxP*, and γ*LacZ* mice. We mated α*1MV* and α*CR-loxP* mutant mice for *Pcdha*^*del*^*;* β*1-loxP* and β*22-loxP* mutant mice for *Pcdhb*^*del*^; β*22-loxP* and γ*LacZ* mutant mice for *Pcdhg*^*del*^*;* α*1MV* and β*22-loxP* mutant mice for *Pcdhab*^*del*^*;* β*1-loxP* mutant mice and γ*LacZ* for *Pcdhbg*^*del*^; and α*1MV* mutant mice and γ*LacZ* for *Pcdhabg*^*del*^. In addition, we generated male mice carrying distinct combinations of the *Pcdh*-cluster mutant alleles (commonly derived from TT2 ES cells) and the *Sycp-Cre* transgene (C57BL/6 genetic backgound) (Noguchi et al., 2009). These mice were crossed with C57BL/6 females, and the pups were genotyped by PCR. The primer sequences used for genotyping are shown in Supplementary Table S1. Their homozygous and heterozygous mutants were commonly obtained and maintained by crossing between their heterozygous male and female.

### Generation of *TG^*taf7*^* transgenic mice

A 20-kb DNA fragment containing the *TAF7* gene, which is located upstream of the *Pcdh*γ cluster, was isolated from the mouse BAC RP23-440M12 by BAC modification methods and used as a transgene to generate *TG*^*taf7*^ transgenic mice. The transgene was injected into eggs of C57BL/6 mice, and the eggs were implanted into the uterus of ICR mice, using standard protocols (Gong et al., 2003). The genotyping primers of T1 and T2 are shown in Supplemental Table S1.

### RT-PCR and immunoblot analyses

RT-PCR and immunoblot analyses were performed as described previously (Hasegawa et al., 2008). Quantitative RT-PCR analysis was performed as described previously (Yokota et al., 2011). The primer sequences are shown in Supplementary Table S1.

### Immunohistochemistry

Immunohistochemistry was performed as described previously (Hasegawa et al., 2008) with the following antibodies: Anti-calretinin (Chemicon); anti-calbindin (Sigma); anti-ChAT (Chemicon); anti-NeuN (Chemicon); anti-Chx10 (Santa Cruz); anti-FoxP2 (Sigma); anti-GFAP (Sigma); anti-MAP2 (Millipore); anti-Parvalbumin (Abcam); anti-Syntaxin (Stressgen); anti-Tuj1 (Covance); anti-VGluT1 (Chemicon); anti-VGluT2 (Chemicon); anti-VGAT (Frontier Institute); anti-Cleaved-caspase-3 (Cell Signaling Technology); anti-pan axonal-neurofilament (SMI312, Covance); anti-neurofilament200 (Sigma); anti-Pcdhα (produced by CBSN); and anti-Pcdhγ (Murata et al., 2004). Neuro Trace 435 (Molecular Probes) was used as a Nissl stain.

### Immunoprecipitation analysis

Mouse brains (E18.5-P0) were homogenized in homogenization buffer (0.32 M sucrose, 10 mM Tris-HCl pH7.5, and 2 mM EDTA) with protease inhibitors (Complete Mini protease inhibitors, Roche). The homogenate was centrifuged at 800 × g at 4°C for 10 min. The supernatant was centrifuged at 20,000 × g at 4°C for 30 min. The pellet was lysed with cell-lysis buffer (20 mM Tris-HCl pH 7.5, 150 mM NaCl, 1 mM EDTA, 1% Triton X-100, 0.25% sodium deoxycholate, and Complete Mini protease inhibitors) and centrifuged at 20,000 × g at 4°C for 30 min. The supernatant was used for immunoprecipitation.

For immunoprecipitation assays, lysates of brain tissue containing 3.5 mg of protein were incubated with antibodies (rabbit anti-Pcdhβ2, guinea pig anti-Pcdhα, and guinea pig anti-Pcdhγ) and Protein A-Sepharose beads (GE Healthcare) for 2 h at 4°C. The beads were washed twice with cell-lysis buffer and twice with Phosphate buffered saline (PBS). The bound proteins were eluted by boiling the beads in SDS-PAGE sample buffer (50 mM Tris-HCl pH 6.8, 2% SDS, 2% v/v 2-mercaptoethanol, and 5% glycerol), separated by SDS-PAGE, and immunoblotted with a guinea pig anti-PcdhγCR antibody (produced by CBSN), mouse anti-PcdhαCR antibody (4F8)(Katori et al., 2009), or rabbit anti-Pcdhβ2 antibody (Sigma Aldrich).

### Electrophysiology

The spinal cord from E18.5 embryos was removed by ventral laminectomy as previously described (Saito et al., 2010). The isolated spinal cord was placed in a recording chamber perfused with oxygenated Ringer's solution (118.4 mM NaCl, 3 mM KCl, 2.52 mM CaCl_2_, 1.25 mM MgSO_4_, 25 mM NaHCO_3_, 1.18 mM KH_2_PO_4_, and 11.1 mM D-glucose aerated with 5% CO_2_ in O_2_) at room temperature. Recording of the lumbar VR was done by a glass suction electrode placed in close proximity to the exit point of the root. VR signals were amplified 10,000 times and bandwidth filtered at 10–3000 Hz with a DAM 50 AC amplifier (WPI, Sarasota, FL). Signals were digitized (5 kHz) and recorded to a hard disk with Clampex 10.2 software (Molecular Devices). All drugs [NMDA and 5-HT (Sigma, St. Louis, MO)] were dissolved in Ringer solution and bath applied to the preparation. Locomotor-like rhythmic activity was evoked by bath application of NMDA in combination with 5-HT as previously described (Nishimaru et al., 2011).

### Imaging

Fluorescent images were captured on an FV1000 confocal microscope (Olympus, Japan), a BZ9000 microscope (Keyence, Japan), or a BZ-X710 microscope (Keyence, Japan). Images were prepared for printing with Adobe Photoshop Elements Editor. Neurons were counted manually in blinded conditions. To measure synaptic density onto MNs, images were obtained with an X60 oil objective lens on an Olympus FV1000 confocal microscope with X3.8 digital zoom. All images for comparisons were blinded to the condition. We used KEYENCE's original algorithm to quantify the olfactory-bulb data.

### Statistical analysis

Statistical analyses were conducted using Prism version 5 (GraphPad Software, Inc.) to apply one-way Analysis of variance (ANOVA) and *post-hoc* Tukey tests. Values shown in graphs are the mean ± SEM.

## Results

### Mice with various combinations of *Pcdh-*cluster deletions (Δα, Δβ, Δγ, Δαβ, Δβγ, and Δαβγ mutants)

**Figure 1:**
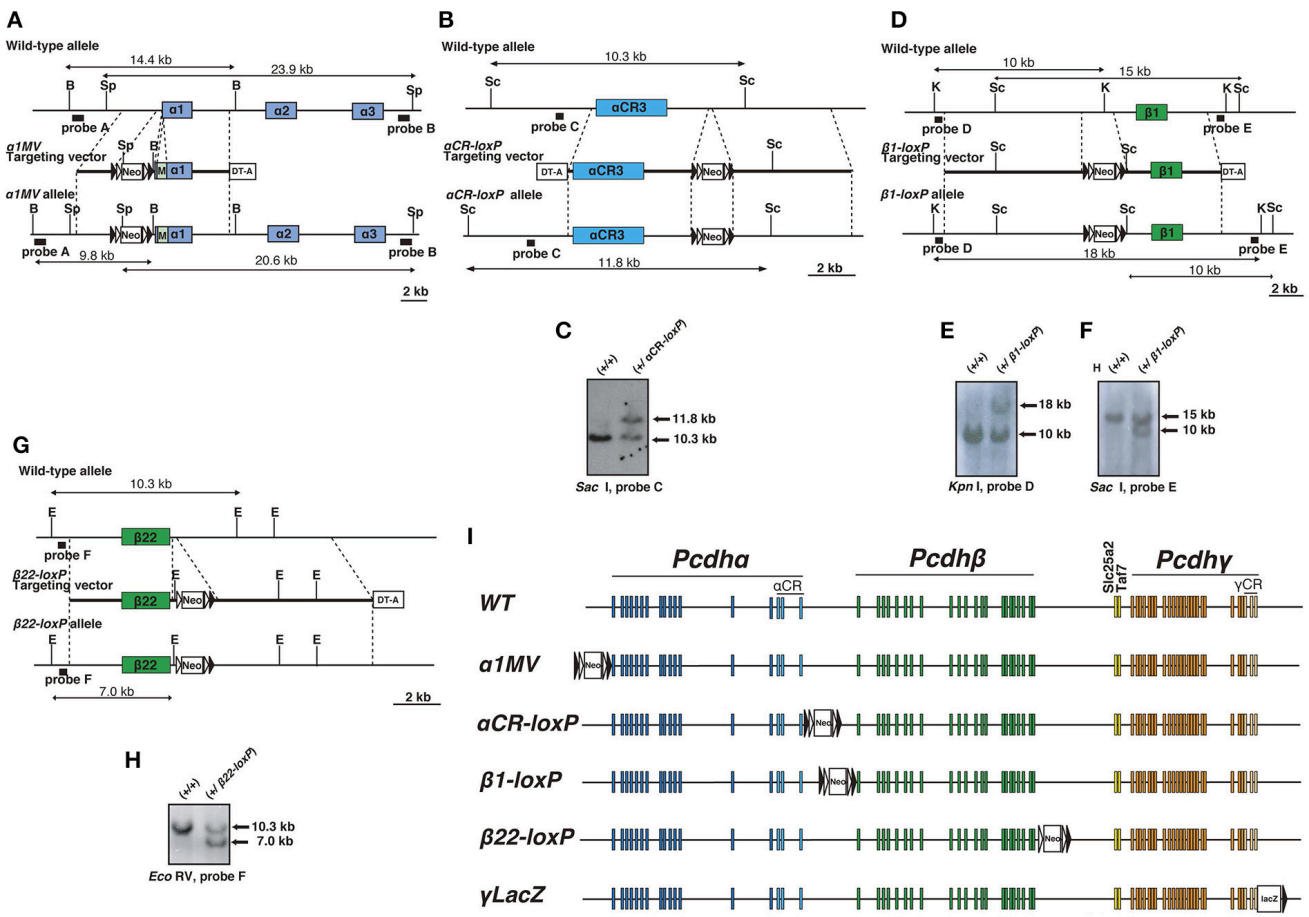
**Generation of mice with *loxP*-site insertions**. **(A)** Generation of α*1MV* mutant mice. Diagram of the α*1MV* targeting constructs. M: Myc-tagged-Venus fluorescent protein gene. B: *Bam*HI; Sp, *Spe*I. **(B,C)** Generation of α*CR-loxP* mutant mice. **(B)** Diagram showing the α*CR-loxP* targeting constructs. Sc: *Sac* I. **(C)** Southern blot of homologous recombinant ES cells digested by *Sac* I, with Probe C. **(D–F)** Generation of β*1-loxP* mutant mice. **(D)** Diagram showing the β1-loxP targeting constructs. K: *Kpn* I; Sc: *Sac* I. Southern blots of homologous recombinant ES cells **(E)** digested by *Kpn* I, with Probe D or **(F)** digested by *Sac* I, with Probe E. **(G,H)** Generation of β*22-loxP* mutant mice. **(G)** Diagram showing the β*22-loxP* targeting constructs. E: *Eco*RV. **(H)** Southern blot of homologous recombinant ES cells digested by *Eco*RV with Probe F. **(I)** Genetic structures of mice with *loxP*-site insertions. The *loxP* sites were inserted into the following regions: 5′of the *Pcdh*α cluster in α*1MV* mice, 3′ of the Pcdhα cluster in α*CR-loxP* mice, 5′ of the *Pcdh*β cluster in β*1-loxP* mice, 3′ of the *Pcdh* β cluster in β*22-loxP* mice, and 3′ of the *Pcdh*γ cluster in γ*LacZ* mice. Filled and open triangles represent *loxP* and *frt* sites, respectively.

**Figure 2:**
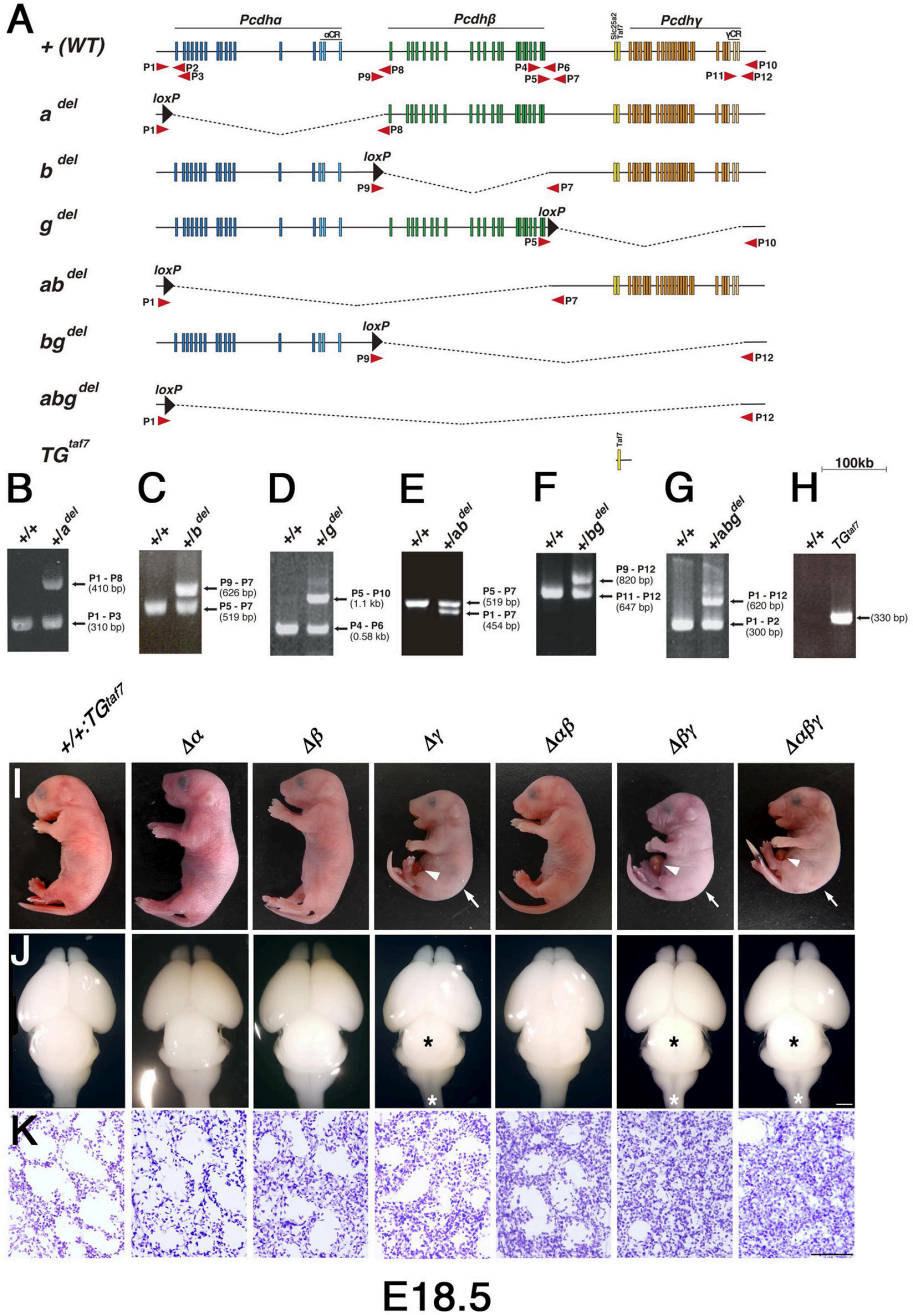
**Generation of cluster-deletion mutant mice by Cre-dependent recombination between mice with trans-allelic *loxP* sites and *TG*^*taf7*^ BAC-transgenic mice. (A)** Genetic structures of cluster-deletion mutant mice and *TG*^*taf7*^ mice. The cluster-deletion mutants *Pcdha*^*del*/*del*^*, Pcdhb*^*del*/*del*^*, Pcdhg*^*del*/*del*^*:TG*^*taf7*^*, Pcdhab*^*del*/*del*^*, Pcdhbg*^*del*/*del*^*:TG*^*taf7*^, and *Pcdhabg*^*del*/*del*^*:TG*^*taf7*^ were generated by Cre-induced meiotic recombination between α*1MV*, α*CR-loxP*, β*1-loxP*, β*22-loxP*, and γ*LacZ* mice (Figure 1). *TG*^*taf7*^ mice were generated by microinjecting a Bacterial Artificial Chromosome (BAC) containing the *Slc25a2* and *TAF7* genes. Arrows indicate primer positions used for genotyping. **(B–H)** Genotyping of the cluster-deletion mutants and *TG*^*taf7*^ transgenic mice. **(B)** *a*^*del*^*/a*^*del*^, **(C)** *b*^*del*^*/b*^*del*^, **(D)** *g*^*del*^*/g*^*del*^*:TG*^*taf7*^, **(E)** *ab*^*del*^*/ab*^*del*^, **(F)** *bg*^*del*^*/bg*^*del*^*:TG*^*taf7*^, **(G)** *abg*^*del*^*/abg*^*del*^*:TG*^*taf*7^, and **(H)** *TG*^*taf7*^. **(I)** Gross phenotypes of E18.5 cluster-deletion mutant mice. The Δγ, Δ*βγ*, and Δ*αβγ* mutants had a hunched posture (arrows) and umbilical hernia (arrowheads) in most cases. **(J)** Whole brains of E18.5 cluster-deletion mutant mice. The three Pcdhγ-deletion lines had a smaller midbrain (black asterisks) and thinner medulla and spinal cord (white asterisks). **(K)** Nissl-stained lungs at E18.5. Compared to the Δγ mutant, the lungs of the double-deletion (Δ*βγ*) and triple-deletion (Δ*αβγ*) mutants were more compact and had smaller alveolar spaces due to shallow breathing; these pups survived less than 1 h after birth. Bars: 1 mm in **(J)**; 100 μm in **(K)**.

To create various deletion and duplication alleles, mice carrying each of the targeted *Pcdh* alleles were bred with *SyCP-Cre* transgenic mice, in which Cre recombinase is expressed in meiosis. Heterozygous males containing various combinations of *Pcdh* alleles and *SyCP-Cre* were mated to wild-type C57BL/6J females. We detected the germline transmission of all 12 *loxP*-recombination alleles—*Pcdha*^*del*^*, Pcdha*^*dup*^*, Pcdhb*^*del*^*, Pcdhb*^*dup*^*, Pcdhg*^*del*^*, Pcdhg*^*dup*^*, Pcdhab*^*del*^*, Pcdhab*^*dup*^*, Pcdhbg*^*del*^*, Pcdbg*^*dup*^*, Pcdhabg*^*del*^, and *Pcdhabg*^*dup*^—in the offspring (Figure 1). To investigate the function of each *Pcdh* cluster, we produced the homozygous cluster-deletion mutants *Pcdha*^*del*/*del*^*, Pcdhb*^*del*/*del*^*, Pcdhg*^*del*/*del*^*, Pcdhab*^*del*/*del*^*, Pcdhbg*^*del*/*del*^, and *Pcdhabg*^*del*/*del*^ by crossing heterozygous mutants (Figures 2A–H). Homozygous mutants with a single *Pcdh*α deletion (Δα), a single *Pcdh*β deletion (Δβ), or double *Pcdh*α and *Pcdh*β deletion (Δ*αβ*) were born at near-Mendelian frequency, survived, and were fertile. On the other hand, we were initially surprised not to recover any *Pcdhg*^*del*^ mice, based on prior published work indicating that they survive until birth (Wang et al., 2002; Hambsch et al., 2005). We realized that this could be due to loss of the *TAF7* gene in this meiotically recombined line, given that *TAF7*-deficient mice are known to be early embryonic lethal (Gegonne et al., 2012). Therefore, to rescue the *TAF7* gene, which is located between the *Pcdh*β and *Pcdh*γ clusters, we produced a *TG*^*taf7*^ transgenic mouse line using a BAC plasmid of a 20-kb region between the *Pcdh*β and *Pcdh*γ clusters that included the *TAF7* gene. By crossing the *TG*^*taf7*^ transgenic mice and heterozygous *Pcdhg*^*del*/+^ mutants, we obtained homozygous *Pcdhg*^*del*/*del*^ mutants with the *TG*^*taf7*^ transgene, *Pcdhg*^*del*/*del*^*:TG*^*taf7*^ (Δγ) mice. These mice were born alive and exhibited the same phenotypes of a hunched posture and repetitive limb tremors (Supplementary Movie 1) as previously reported *Pcdh*γ-knockout mice (Wang et al., 2002), indicating that the *TG*^*taf7*^ transgene completely rescued the early embryonic lethality caused by the TAF7 deficiency. We also produced the homozygous mutants *Pcdhbg*^*del*/*del*^*; TG*^*taf7*^ (Δ*βγ*) and *Pcdhabg*^*del*/*del*^*; TG*^*taf7*^ (Δ*αβγ*) by crossbreeding with the *TG*^*taf7*^ transgenic mice.

### Δβγ- and Δαβγ-deficient mutants

In our first attempt to investigate cooperative functions in the *Pcdh* clusters, we generated Δγ, Δ*βγ*, and Δ*αβγ* mutants. The Δ*βγ* and Δ*αβγ* mutants (Figure 2I) had a normal heartbeat but exhibited acromphalus (arrowheads), a hunched posture (arrows), shallow breathing, little movement, and no response to any touch or physical stimuli; these mice died shortly after birth (Supplementary Movies 2, 3). The pups from these three lines similarly had a small midbrain and thin medulla and spinal cord (Figure 2J, asterisks). Histological examination revealed that the Δ*βγ* and Δ*αβγ* mutants had compact lung tissues with small alveolar spaces caused by shallow, irregular breathing (Figure 2K), but no obvious defects in any other internal organs (data not shown). These neonatal-lethal phenotypes were distinct from the repetitive-tremor phenotype seen in the Δγ single mutant, which had almost normal lung tissue, suggesting that the *Pcdh*β and *Pcdh*γ clusters share some overlapping functions.

### *Pcdh* expression in various *Pcdh*-cluster deletion mutants

**Figure 3:**
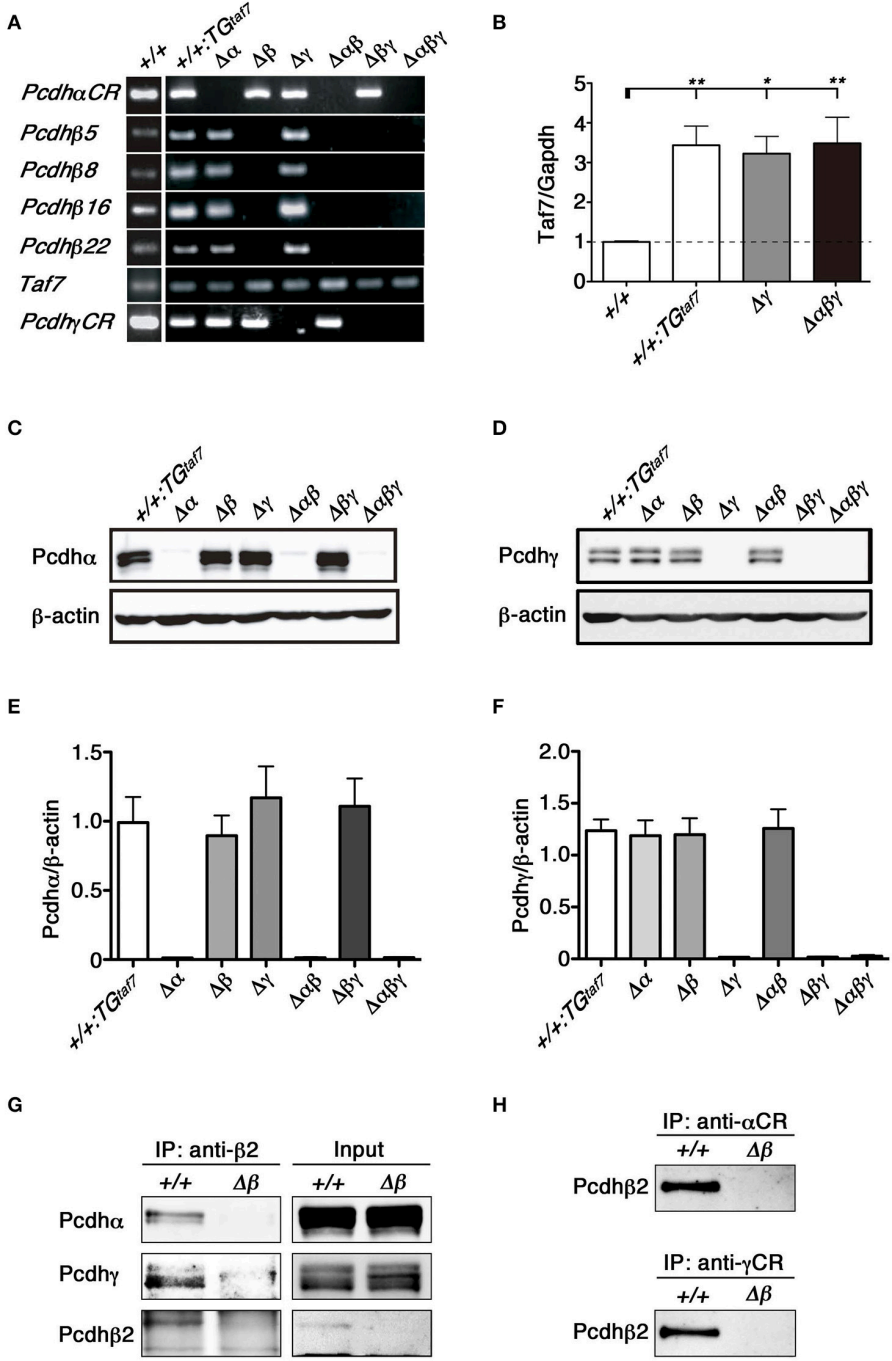
**RT-PCR, immunoblot analysis and the co-association of Pcdhα, Pcdhβ, and Pcdhγ *in vivo***. **(A)** RT-PCR analysis of the whole brain of various mutant mice at E18.5. **(B)** Quantification of *TAF7* transcripts of representative mutant animals at E18.5 by quantitative RT-PCR analysis. RT-PCR reactions in absence of reverse transcriptase showed the samples had no genomic DNA contaminants (data not shown). *N* = 4–5 animals per genotype. Error bars represent SEM. ^**^P < 0.01 and ^*^P < 0.05 by one-way ANOVA and *post-hoc* Tukey tests. **(C,E)** E18.5 brain lysates were immunoblotted with **(C)** anti-Pcdhα and **(E)** anti-Pcdhγ antibodies. **(D,F)** Quantification of **(D)** Pcdhα and **(F)** Pcdhγ proteins. *N* = 4 animals per genotype. Error bars represent SEM. There was no signifcance among these genotypes by one-way ANOVA and *post-hoc* Tukey tests. **(G,H)** The lysates of crude membrane fractions from the E18.5-P0 mouse brain were co-immunoprecipitated with **(G)** anti-Pcdhβ2, **(H)** anti-Pcdhα, or anti-Pcdhγ antibodies. The precipitates were immunoblotted with anti-Pcdhβ2, anti-Pcdhα, and anti-Pcdhγ.

We first confirmed that each deletion mutant was a complete null-mutant strain by examining the expression of clustered *Pcdh* genes in each line by reverse transcriptase (RT)- polymerase chain reaction (PCR). As expected, *Pcdh*β genes disappeared completely in the brains of Δβ, Δ*βγ*, and Δ*αβγ* mutants, and no *Pcdh* transcripts were detected in the brains of Δ*αβγ* mutants (Figure 3A).We next performed quantitative RT-PCR analysis to determine the relative expression level of *TAF7* transcripts in the following mutants, WT, +*/*+*; TG*^*taf7*^*, Pcdhg*^*del*/*del*^*; TG*^*taf7*^ (Δγ) and *Pcdhabg*^*del*/*del*^*; TG*^*taf7*^ (Δ*ab*γ). As a result, in the *TAF7* transgenenic animals, the expression levels of *TAF7* was significantly increased to more than three times of the level in the WT animal (Figure 3B). Then we performed all experiments by using the +*/*+*; TG*^*taf7*^ mice as controls unless othewise noted.

To confirm the deletion of Pcdhα and Pcdhγ at the protein level, brain lysates from each mutant strain were immunoblotted using anti-Pcdhα and -Pcdhγ antibodies raised against the constant regions shared by all members of the Pcdhα and Pcdhγ families. We did not detect any bands corresponding to Pcdhα proteins in the brains of Δα, Δ*αβ*, or Δ*αβγ* mutants (Figure 3C). Similarly, we confirmed the deletion of Pcdhγ proteins in the brains of Δγ, Δ*βγ*, and Δ*αβγ* mutants (Figure 3E). Measurement of the Pcdhα and Pcdhγ protein expression levels confirmed that the deletion of one cluster did not alter the expression levels of the others (Figures 3D,F). These results confirmed that each deletion mutant was a complete null-mutant mouse strain.

**Figure 4:**
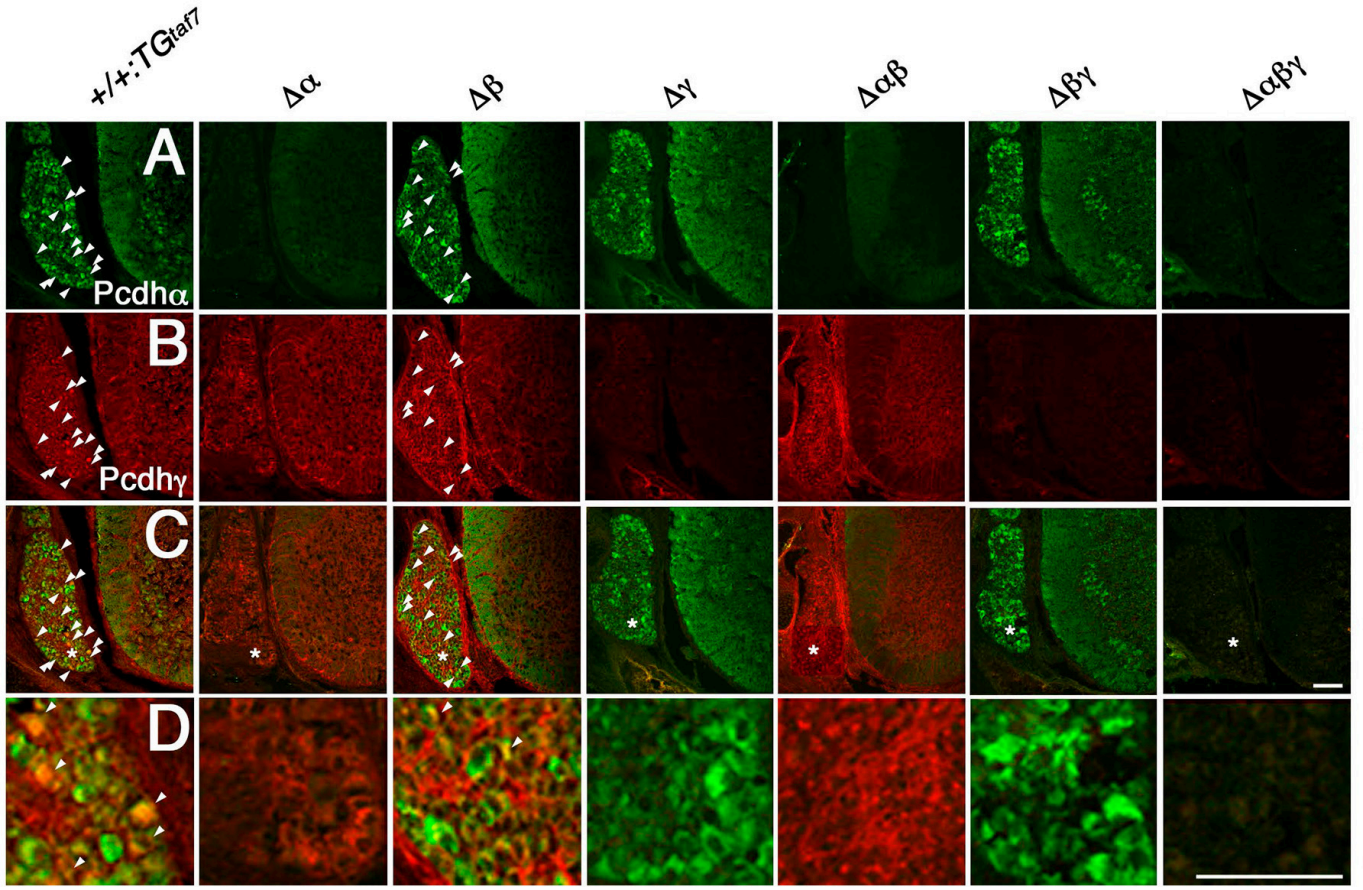
**Immunohistochemistry in the spinal cord**. **(A–D)** Pcdhα and Pcdhγ proteins were widely distributed in sections from the E16.5 cervical spinal cord. **(A)** Many DRG neurons were differentially stained by an anti-Pcdhα antibody. Moderate Pcdhα immunoreactivity was extensively detected throughout the ventral cord. In contrast, **(B)** Pcdhγ immunoreactivity was broader than that of Pcdhα, and was distributed in the DRG and spinal cord. **(C)** Double-staining with anti-Pcdhα and anti-Pcdhγ antibodies revealed that subpopulations of DRG sensory neurons co-expressed Pcdhα and Pcdhγ (arrowheads). **(D)** Magnified views of the regions (^*^) were shown. There was no Pcdhα or Pcdhγ immunoreactivity in the Δα and Δγ spinal cords, respectively, confirming that each deletion mutant was a complete null-mutant strain. Bars: 100 μm.

*Pcdh*α and *Pcdh*γ mRNA and proteins are highly expressed in the embryonic spinal cord (Carroll et al., 2001; Wang et al., 2002). Here we examined the distribution pattern of Pcdhα and Pcdhγ in the spinal cord. As expected, sections from homozygous Δα, Δ*αβ*, and Δ*αβγ* mutants had no Pcdhα immunoreactivity (Figure 4A), and no Pcdhγ immunoreactivity was detected in sections from homozygous Δγ, Δ*βγ*, or Δ*αβγ* mutants (Figure 4B). In the E16.5 cervical spinal cord, many dorsal root ganglion (DRG) sensory neurons were stained by the anti-Pcdhα antibody, although only some DRG neuron subpopulations were intensely stained by the anti-Pcdhγ antibody. Moderate Pcdhα immunoreactivity was detected throughout the ventral spinal cord, where there are many interneuron populations and motor neurons (MNs) (Figure 4A). In contrast, Pcdhγ immunoreactivity was detected more broadly, in both the DRG and the spinal cord (Figure 4B). Double-staining with anti-Pcdhα and anti-Pcdhγ antibodies revealed that subpopulations of DRG sensory neurons co-expressed Pcdhα and Pcdhγ (Figures 4C,D). These experiments revealed the distribution of Pcdhα and Pcdhγ proteins in the spinal cord, and confirmed the null mutation of each mutant strain and the specificities of the anti-Pcdhα and -Pcdhγ antibodies.

### Pcdhα, Pcdhβ, and Pcdhγ proteins cooperatively associate as a hetero-oligomer *in vivo*

We previously showed that the Pcdhα and Pcdhγ proteins associate *in vivo* (Murata et al., 2004). The protein complexes between Pcdhβ and Pcdhγ proteins are shown by proteomics analysis (Han et al., 2010). To examine protein interactions between Pcdhα and Pcdhβ, or between Pcdhγ and Pcdhβ, we immunoprecipitated E18.5-P0 brain lysates of +*/*+ and single Δβ mutant mice with an anti-Pcdhβ2 antibody (Figure 3G). Conversely, we then examined whether Pcdhβ could be co-immunoprecipitated with an anti-Pcdhα or anti-Pcdhγ antibody (Figure 3H). Immunoprecipitation analyses revealed that the Pcdhβ2 proteins interacted with both Pcdhα and Pcdhγ proteins, and that these three *Pcdh* clusters could cooperatively function as an oligomer at the protein level *in vivo*.

### Olfactory-axon abnormalities were present in all *Pcdh* mutant lines

**Figure 5:**
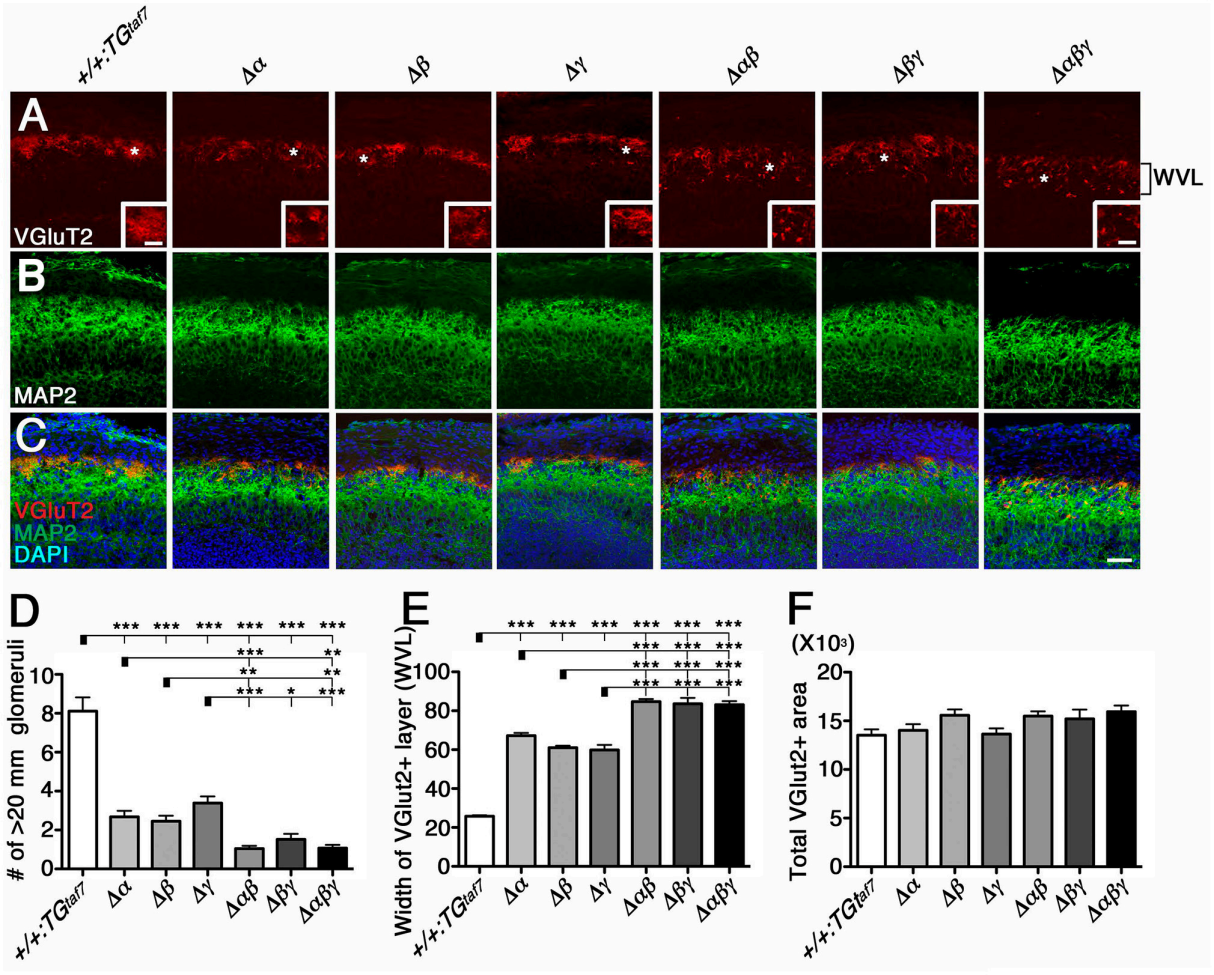
**Cooperative function of the *Pcdh*α, *Pcdh*β, and *Pcdh*γ clusters in olfactory-circuit formation during prenatal development**. **(A–C)** In E18.5 olfactory bulbs, double-staining with VGluT2 **(A)** and MAP2 **(B)** showed abnormal glomerular structure even in single-deletion mutants, with more extensive disruption in double- or triple-deletion mutants. Inset in **(A)** is its magnified view of the ^*^ region. **(D)** The number of glomerular-like structures with a diameter > 20 μm. **(E)** The width of the VGlut2^+^ layer (WVL), presynaptic axon terminals of olfactory sensory neurons in the olfactory bulb was significantly greater in the Δ*αβ* and Δ*βγ* double mutants than in the single mutants (Δα, Δβ, and Δγ). **(F)** The total VGlut2^+^ area was similar in all mutants. *N* = 4–6 animals per genotype. Error bars are SEM. ^***^P < 0.001, ^**^P < 0.01, and ^*^P < 0.05 by one-way ANOVA and *post-hoc* Tukey tests. Bars: 20 μm in **(A)** and 50 μm in **(C)**.

We previously described the olfactory phenotypes of homozygous *Pcdha*^*dCR*/*dCR*^ pups, which have malformed glomerular structures and disrupted olfactory sensory-axon targeting in the olfactory bulb (Hasegawa et al., 2012). Therefore, we next examined olfactory-axon abnormalities in our six lines of homozygous cluster-deletion mutants—the single-deletion Δα, Δβ, and Δγ, double-deletion Δ*αβ* and Δ*βγ*, and triple-deletion Δ*αβγ* mutants. We first examined phenotypes previously described in the *Pcdh*α-deletion mutant. Glomerular structures begin forming on embryonic day (E) 15–16, when olfactory sensory axons and mitral cell dendrites both contribute to their formation (Blanchart et al., 2006). For this analysis, we immunostained E18.5 olfactory bulbs with anti-VGluT2 and anti-MAP2 (Figures 5A–C). We found that the glomerular structure appeared abnormal not only in single Δα mutants, but also in single Δβ and Δγ mutants, and that the disruption of olfactory-axon projection patterns appeared more extensive in the Δ*αβ* and Δ*βγ* double-deletion and Δ*αβγ* triple-deletion mutants than in the single-deletion mutants (Δα, Δβ, and Δγ) (Figure 5A, WVL). We next counted any glomerular-like structures with a diameter >20 μm. We defined a glomerulus as a spherical structure of neuropils surrounded by the nuclei of periglomerular cells, as revealed by 4′,6-diamidino-2-phenylindole (DAPI) staining. Consistent with our initial observations, all six mutant lines had significantly fewer glomeruli with a diameter >20 μm than did +*/*+*:TG*^*taf7*^ control mice (Figure 5D). Next, we measured the width of the VGlut2^+^ layer (presynaptic axon terminals of olfactory sensory neurons) in olfactory bulbs from each genotype. The VGlut2^+^ layer (Figure 5A, WVL) was significantly wider in the Δ*αβ* and Δ*βγ* double mutants than in the single mutants (Δα, Δβ, and Δγ) (Figure 5E); however, the total VGlut2^+^ area was essentially the same in all lines of mutant animals (Figure 5F). The number of glomeruli with a diameter >20 μm in the WVL of triple Δ*αβγ* mutants was similar to that in the double mutants Δ*αβ* and Δ*βγ*. Thus, all three clusters—*Pcdh*α, *Pcdh*β, and *Pcdh*γ—had unique and overlapping functions for olfactory glomerular formation during prenatal development, suggesting that glomerular formation and the axon targeting of olfactory sensory neurons are cooperatively regulated by all three *Pcdh* clusters. These findings showed that the *Pcdh*α, *Pcdh*β, and *Pcdh*γ families are all required for proper olfactory-circuit formation, and that the *Pcdh* clusters have distinct and cooperative functions. Moreover, it is highly possible that the molecular diversity of Pcdh-family proteins is functionally significant.

### Abnormal Ia primary afferents in the ventral horn are caused exclusively by the deletion of the *Pcdh*γ cluster

**Figure 6:**
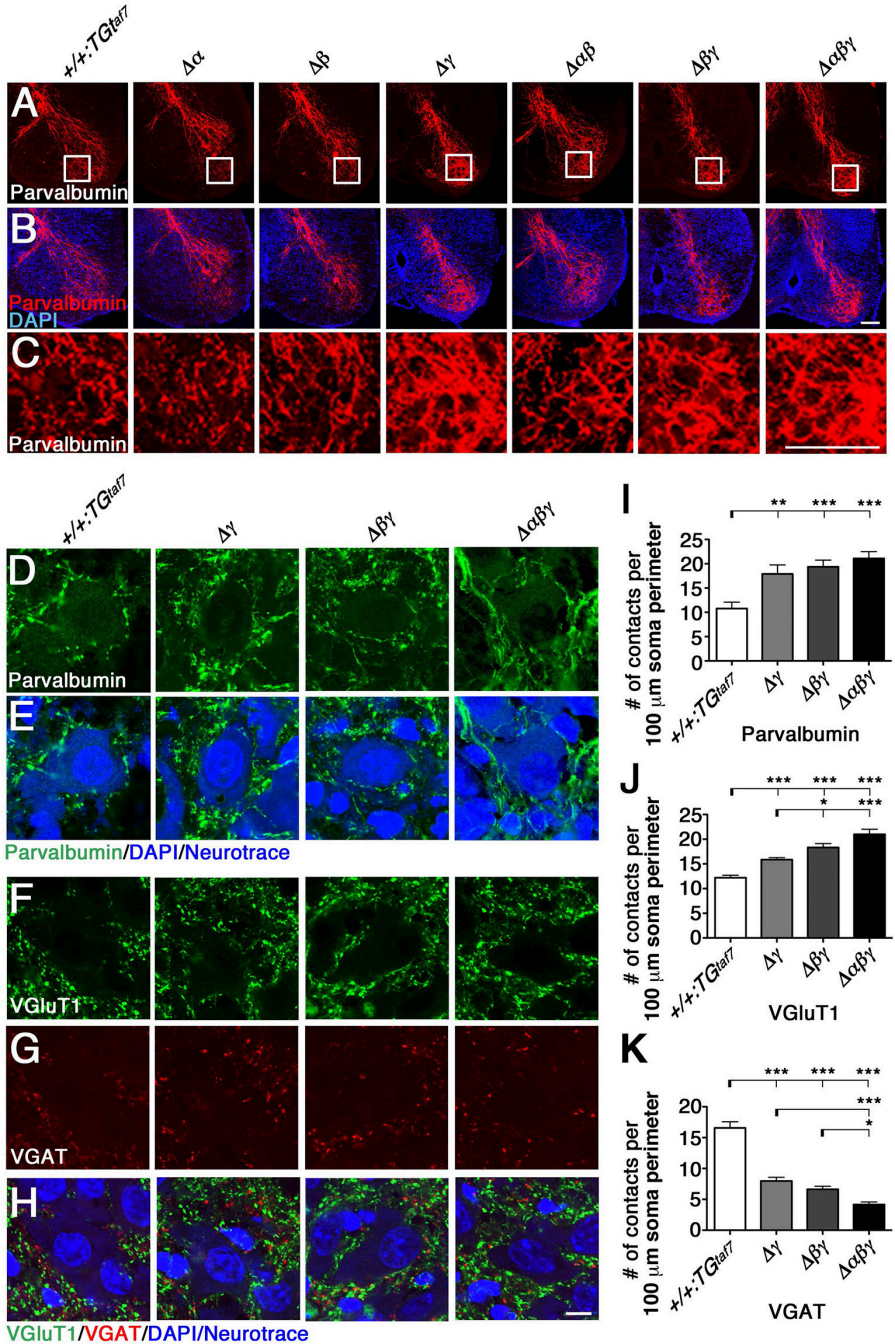
**Terminal arborization of parvalbumin^+^ Ia afferents surrounding motor pools and alteration of the synaptic density of MNs**. **(A–C)** Ia afferent terminals (Parvalbumin^+^; red) in the ventral horn were severely disorganized in the single Δγ, double Δ*βγ*, and triple Δ*αβγ* mutants, but appeared normal in the three lines that retained the *Pcdh*γ cluster. Regions surrounded by frame lines are magnified in **(C)** respectively. **(D–K)** Altered MN synaptic densities in **(D)** Parvalbumin^+^ (green). **(F)** VGluT1^+^ (green) and **(G)** VGAT^+^ (red) contacts. **(E,H)** MNs were counterstained with DAPI (blue) and Neurotrace (blue) to identify the nuclei and cell bodies, respectively. **(I–K)** Number of Parvalbumin^+^, VGluT1^+^ and VGAT^+^ contacts per 100 μm soma perimeter. Consistent with the neuron-death phenotype, the extent of alteration of synaptic contacts onto MNs was correlated with the number of deletions of Pcdhα, Pcdhβ, and Pcdhγ clusters. *N* = 3 animals per genotype in **(I)**, *N* = 4–6 animals per genotype in **(J,K)**. Error bars represent SEM. ^***^P < 0.001, ^**^P < 0.01, ^*^P < 0.05 by one-way ANOVA and Tukey's *post-hoc* test. Bars: 100 μm in **(B,C)** and 10 μm in **(H)**.

We next analyzed the abnormal axon projections into the spinal cord, as previously reported in the *Pcdh*γ-deletion mutant. The *Pcdh*γ cluster is required for Ia afferent terminal formation in the spinal cord, and Pcdhγ mutants exhibit severe disorganization of the Ia proprioceptive afferent terminals of DRG neurons onto MNs (Prasad and Weiner, 2011). The same clumped-axon abnormality is also observed in γ*C* TKO mutants, which lack only the γ*C3*, γ*C4*, and γ*C5* genes (Chen et al., 2012). Thus, it is highly likely that the Pcdhγ cluster is largely responsible for appropriate Ia primary afferent projections in the spinal cord. To determine whether the *Pcdh*α and *Pcdh*β clusters also contribute to the formation of Ia primary afferent circuits, we stained E18.5 lumbar spinal cords from the various mutants with an anti-Parvalbumin antibody (Figures 6A–C). We found that the Ia afferent terminals in the three lines of mutants that retained the *Pcdh*γ cluster appeared normal. On the other hand, the Ia afferent terminals in the ventral horn of the Δγ, Δ*βγ*, and Δ*αβγ* mutants were severely disorganized. Further analysis revealed that centrally projecting Parvalbumin^+^ Ia axon projections were equally distributed around MN pools in control animals, but appeared more aggregated, with higher-density signals closely surrounding the MNs in all of the Δγ, Δ*βγ*, and Δ*αβγ* mutant animals (Figures 6C–E). Quantification of the density of Parvalbumin-stained Ia terminals per MN concluded that of the *Pcdh* clusters, only the *Pcdh*γ cluster (probably the triple γC-type isoforms in particular) is essential for the normal development of Ia afferent axons onto the MNs (Figure 6I). Taken together, these observations indicate that the *Pcdh*γ cluster has unique function in establishing the terminal arborization of Ia afferent axons.

### Altered excitatory and inhibitory synaptic inputs onto MNs

**Figure 7:**
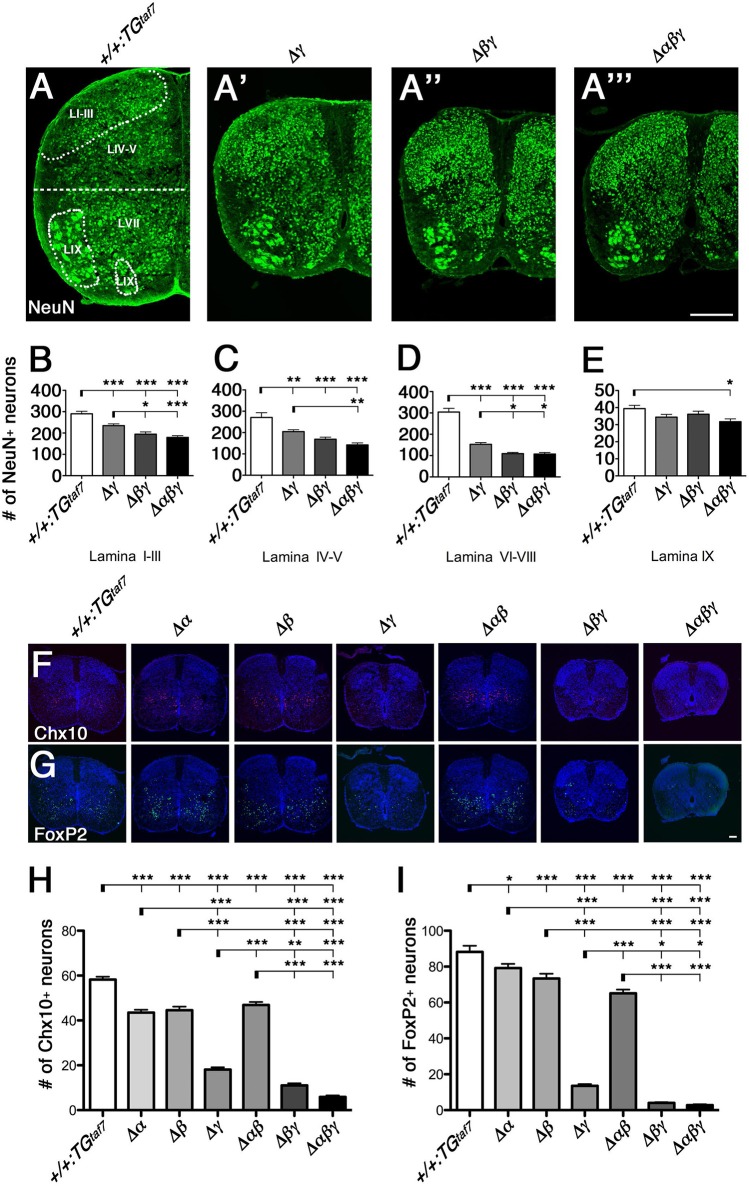
**Neuronal death in the spinal cord. (A–A”')** Surviving NeuN^+^ neuronal populations were counted in four demarcated regions (Lamina I–III, IV–V, VI–VIII, and IX) in the E18.5 hemi cords of control and Pcdhγ-mutant mice. **(B–D)** In all three mutants, the NeuN^+^ populations were severely reduced in the three regions that are primarily abundant in interneurons. **(E)** There was hardly any neuron loss in Lamina IX, which is abundant in MNs. **(F,G)** Representative images of E18.5 spinal cords stained for **(F)** Chx10 and **(G)** FoxP2. **(H,I)** Chx10^+^ and FoxP2^+^ neuron counts in the ventral spinal cord. *N* = 4–6 animals per genotype. Error bars represent SEM. ^***^P < 0.001, ^**^P < 0.01, ^*^P < 0.05 by one-way ANOVA and Tukey's *post-hoc* test. Bars: 200 μm in **(A”')** and 100 μm in **(G)**.

Previous studies describe correlations between neuronal loss and synaptic loss in the spinal cord of the *Pcdh*γ-deletion mutant mouse (Wang et al., 2002; Weiner et al., 2005; Chen et al., 2012). In the present study, we examined synaptic inputs onto MNs by labeling the neurotransmitters glutamate and GABA/glycine with anti-VGluT1 and anti-VGAT, respectively. The VGluT1^+^ proprioceptive primary afferent inputs were increased in Δ*βγ* and Δ*αβγ* mutants compared to Δγ mutants (Figures 6F,J), while the number of VGAT^+^ contacts was less than half of that in control animals (Figures 6G,K). Similar to the NeuN^+^ neuron–loss phenotype (Figures 7B–D), the strength of the phenotype of MN-synapse alteration seen in Δ*βγ* mutants was between that seen in Δγ and Δ*αβγ* mutants (Figures 6J,K). When normalized to the control, the changes in synaptic density for each genotype were as follows: VGluT1: 130% in Δγ, 150% in Δ*βγ*, 172% in Δ*αβγ*; VGAT: 48% in Δγ, 40% in Δ*βγ*, 25% in Δ*αβγ*. The increase in the number of VGluT1^+^ synaptic puncta in the three mutant lines was mainly, but not exclusively, due to an increase in the number of VGluT1^+^ proprioceptive primary afferent inputs.

### Double Δβγ and triple Δαβγ mutants have more severe spinal-cord interneuron apoptosis than do single Δγ mutants

Previous studies showed that at postnatal day 0 (P0), *Pcdh*γ-deletion mutants exhibit massive apoptosis and loss of spinal interneurons (Wang et al., 2002; Weiner et al., 2005; Prasad et al., 2008) and low counts of specific subpopulations of inhibitory and excitatory interneurons, but not of DRG sensory neurons or MNs (Wang et al., 2002; Prasad et al., 2008; Chen et al., 2012). A γ*C* TKO mutant has similar levels and patterns of interneuron loss in the spinal cord (Chen et al., 2012), consistent with the similarity of *Pcdh*γ KO and γ*C* TKO phenotypes; pups from both strains display a hunched posture and limb tremors.

To compare the extent of neuron loss in the single-, double-, and triple-deletion *Pcdh*γ mutants (Δγ, Δ*βγ*, and Δ*αβγ*), we quantified the surviving NeuN^+^ neurons in four specified regions of the E18.5 spinal cord. The cords of the Δ*βγ* and Δ*αβγ* mutant animals had a smaller cross-sectional area than those of the Δγ mutants (Figures 7A'-A”'). In the Δγ-mutant sections, NeuN^+^ neurons were particularly reduced in the ventral horn (LVI–VIII, Figure 7D). On the other hand, the MN pools appeared relatively normal (LIX, Figure 7E). These findings are inconsistent with the high expression of Pcdhα and Pcdhγ proteins in the DRG and MNs (Figures 4A,B), indicating that the level of Pcdh expression in individual neurons and the survivability of each neuron type do not always match. The neuron loss was more extensive in the Δ*βγ* and Δ*αβγ* mutants than in Δγ mutants, in both the superficial dorsal horn (LI–III, Figure 7B) and the ventral horn (LVI–VIII, Figure 7D). The NeuN^+^ neuron counts were lower in the Δ*αβγ* mutants than in the Δ*βγ* mutants, although the difference was not significant (Figures 7B–D). These findings suggest that both the *Pcdh*β and *Pcdh*α clusters have functions that are cooperative with the *Pcdh*γ cluster for neuronal survival. The severity of the neuron-death phenotype was strongly correlated with the number of *Pcdh*-cluster deletions. The survival rates of the NeuN^+^ populations in four locations for all of the strains were as follows: LI–III: +*/*+*:TG*^*taf7*^ 100%, Δγ 80.8%, Δ*βγ* 67.0%, Δ*αβγ* 61.9%. LIV-V: +*/*+*:TG*^*taf7*^ 100%, Δγ 75.6%, Δ*βγ* 62.3%, Δ*αβγ* 52.5%. LVII-VIII: +*/*+*:TG*^*taf7*^ 100%, Δγ 50.3%, Δ*βγ* 35.9%, Δ*αβγ* 35.3%. LIX: +*/*+*:TG*^*taf7*^ 100%, Δγ 87.3%, Δ*βγ* 91.7%, Δ*αβγ* 80.4%.

### *Pcdh*α and *Pcdh*β clusters have distinct and cooperative functions for the survival of spinal interneurons

To determine whether neuronal subpopulations were similarly affected in all six lines of *Pcdh*-deficient mutants, we counted the Chx10^+^ excitatory and FoxP2^+^ inhibitory interneurons in the ventral spinal cord at E18.5. The spinal-cord size in the Δα and Δβ single-deletion and Δ*αβ* double-deletion mutants was indistinguishable from that in control animals (Figures 7F,G). Thus, we speculated that the Pcdhγ cluster plays a key role in the survival of spinal-cord interneurons. However, unexpectedly, we found that the Chx10^+^ excitatory and FoxP2^+^ inhibitory interneurons were also significantly reduced in Δα, Δβ, and Δ*αβ* mutants compared to control mice (Figures 7H,I). Thus, as with the olfactory-bulb phenotypes already mentioned, Pcdhα and Pcdhβ also have distinct functions for the survival of spinal-cord interneurons during prenatal development. A previous study found that in *Pcdh*γ-deletion mutants, the Chx10^+^ and FoxP2^+^ interneuron counts were reduced to 35 and 25% of those in control mice (Prasad et al., 2008; Chen et al., 2012). In the present study, the numbers of Chx10^+^ and FoxP2^+^ neurons were reduced to 31.1 and 15.4% of the control levels in single Δγ mutants, but to 18.9 and 4.5% in double Δ*βγ* mutants (Figure 7). These results indicated that the randomly regulated *Pcdh*β isoforms, but not the *Pcdh*γ*A* and *Pcdh*γ*B* isoforms, have significant functional overlapping with triple *Pcdh*γ*C* isoforms for interneuron survival. In addition, the loss of Chx10^+^ and FoxP2^+^ interneurons was greater in Δ*αβγ* mutants than in Δ*βγ* mutants, although the difference was not significant. Taken together, both *Pcdh*β and *Pcdh*α have overlapping functions with *Pcdh*γ for neuronal survival. The survival rates for Chx10^+^ and FoxP2^+^ neurons in the ventral cord of each deletion mutant were as follows: Chx10^+^: Δα 74.6%, Δβ 76.5%, Δγ 31.1%, Δ*αβ* 80.5%, Δ*βγ* 18.9%, Δ*αβγ* 10.2%. FoxP2^+^: Δα 89.8%, Δβ 83.2%, Δγ 15.4%, Δ*αβ* 73.9%, Δ*βγ* 4.5%, Δ*αβγ* 3.2%.

### Locomotor-circuit malfunction in single Δγ and triple Δαβγ mutants

As the neuronal death was most severe in the ventral cord, we analyzed the central pattern generators (CPGs), which are spinal neuronal circuits within the ventral cord that control locomotion. The functional contribution of each class of interneurons to spinal CPGs is well-studied and the knockout phenotypes of spinal interneurons are loss of left-right coordination (V0), slow rhythm (V1), left-right synchrony at high speed (V2), and unbalanced rhythm (V3). To compare the differences in physiological defects and levels of neuronal death in the spinal cord, we conducted a physiological analysis of the Δγ and Δ*αβγ* mutant animals. We did this because the Δγ and Δ*αβγ* phenotypes were different between the repetitive limb tremors and the little limb movement, respectively.

**Figure 8:**
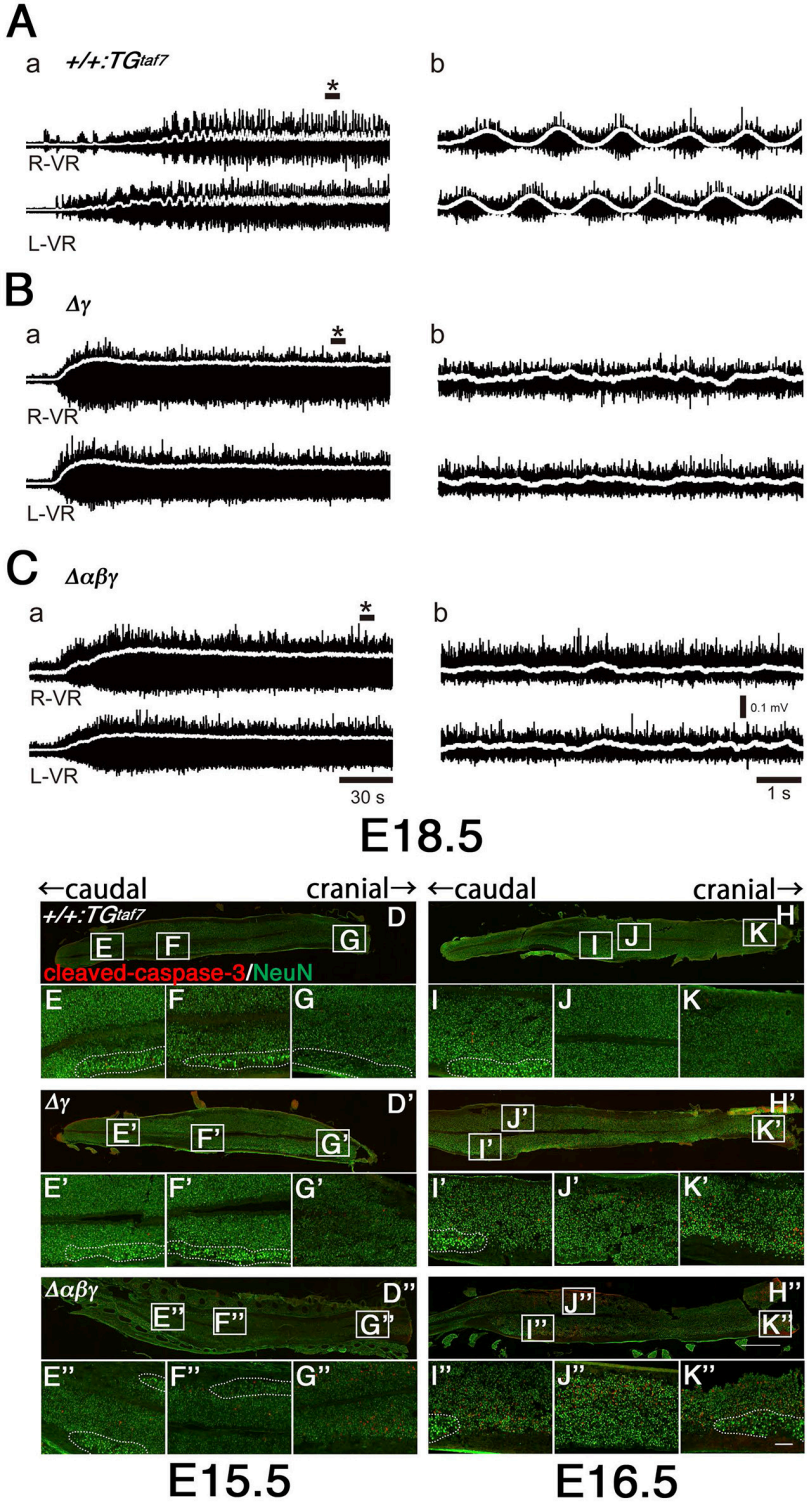
**Abnormal neuron death and the absence of alternating lumbar left-right locomotor-like activity in Δγ and Δαβγ mutants**. **(A)** Normal left-right alternation of locomotor-like activity was recorded after applying NMDA and 5-HT to spinal cords isolated from E18.5 control animals. Recordings show the ventral-root (VR) activity of the second lumbar (L2) segment on the right (R-VR) and left (L-VR) sides; b panels show magnified views of (^*^) in the a panels. **(B,C)** No clear right-left alternating rhythm was observed in the **(B)** Δγ or **(C)** Δ*αβγ* mutant animals. E15.5 **(D–G)** and E16.5 **(H–K)** spinal cords were double-labeled with cleaved-caspase-3 and NeuN. During the embryonic development of locomotor patterns in the spinal cord, abnormal neuron death occurred in the Δγ and Δ*αβγ* mutants, especially in the ventral region around the motor pools, where the locomotor CPGs that govern limb movements during walking are located. All of the MNs within the motor pools (shown by dotted circles) were cleaved-caspase-3^−^ and were probably functional. Bars: 1 mm in **(H”)**; 100 μm in **(K”)**.

The locomotor CPG underlying hindlimb movements during walking is a major motor circuit. We isolated E18.5 spinal cords, induced locomotor-like activity by a bath application of N-methyl-D-aspartic acid (NMDA) and serotonin (5-HT), and recorded electrical activity at the left and right sides of the ventral root (VR) for the second lumbar (L2) segment (Figure 8A–C). In control mice, we detected alternation of the left L2 and right L2 (Figure 8A), while the locomotor-like activity in Δγ and Δ*αβγ* spinal cords lacked right-left alternation (Figures 8B,C). Even high concentrations of NMDA and 5-HT did not induce left-right locomotor-like activity in the Δγ or Δ*αβγ* mutants. This CPG malfunction suggested that the Pcdh clusters are directly involved in forming or maintaining functional CPGs. In mice, the locomotor patterns in the lumbar spinal cord are organized by the late embryonic stages, and the strict left-right alternating patterns are formed at E15.5–18.5 by bilateral interactions between left and right lumbar networks (Branchereau et al., 2000). This time window for establishing the left-right alternation of rhythmic locomotor activity is highly consistent with our findings of abnormal massive interneuron death around the locomotion circuits between E15.5 and E18.5 in the Δγ and Δ*αβγ* mutants (Figures 8D–K”).

### Massive apoptosis in the brainstem of *Pcdh*-deletion mutants

**Figure 9:**
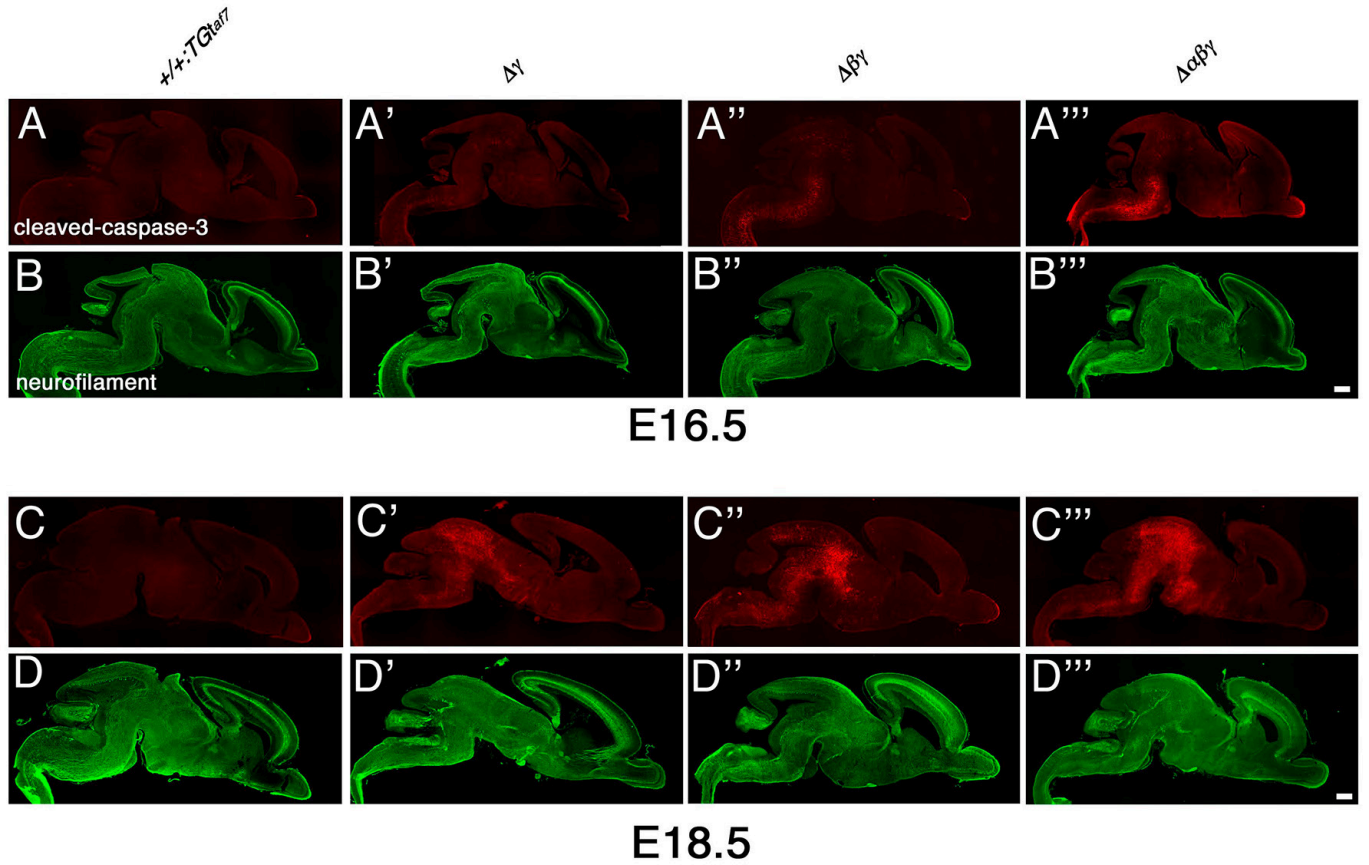
**Massive neuron death in the brainstem of Δγ, Δβγ, and Δαβγ mutants during late embryonic development**. **(A–D”')** Cleaved-caspase-3 (red) and neurofilament (green) double-staining of three mutant strains showed massive neuronal death throughout the brainstem. The severity of the neuronal-death phenotype was correlated with the number of *Pcdh* clusters that were deleted in the strain. Apoptotic cells were mainly detected in the medulla at E16.5, and in the midbrain at E18.5. Thus, neurodegeneration appeared to propagate sequentially from the spinal cord, medulla, and pons to the midbrain during late embryonic development. Compared to control mice, a noticeable and significant reduction in brainstem size was observed in each mutant until E18.5. Bars: 500 μm.

The gross phenotypes of newborn mutants (Figures 2I,J) allowed us to analyze cell death in the Δγ, Δ*βγ*, and Δ*αβγ* mutants. The brainstem regions (including the midbrain, pons, and medulla) and spinal cord of these mutants were smaller than those of control mice (Figure 2J, asterisks). One study briefly described neuronal degeneration in the basal forebrain, thalamus, and medulla in Pcdhγ-KO mutants (Wang et al., 2002). To compare the extent of neuron loss within the whole brain in Pcdh-deletion mutants, we labeled apoptotic cells in E16.5 or E18.5 sagittal whole-brain sections with anti-cleaved-caspase-3 (Figure 9). We found significantly more cleaved-caspase-3^+^ cells in the Δγ mutants than in control animals, and these apoptotic cells were more diffusely scattered throughout the brainstem, including the midbrain (E18.5) and medulla (E16.5), in Δ*βγ* and Δ*αβγ* mutants than in Δγ mutants (Figure 9). As expected, the triple-deletion Δ*αβγ* mutants had the greatest amount of cleaved-caspase-3^+^ cells among the three Pcdhγ-deletion mutants. Thus, the severity of the neuron-loss phenotype was strongly correlated with the number of *Pcdh-*cluster deletions. These results showed that the *Pcdh*α and *Pcdh*β clusters were functionally cooperative with the *Pcdh*γ cluster for neuronal survival in the brainstem. Cooperative function of *Pcdh*α and *Pcdh*β regulation was evident not only in the level and extent of neuronal death, but also in the time course of apoptotic events: Cleaved-caspase-3^+^ signals were markedly noticeable in the E16 medulla of Δ*βγ* but not Δγ mutants. Thus, neurodegeneration appeared to expand sequentially from the spinal cord, medulla, and pons to the midbrain during late embryonic development. However, no cleaved-caspase-3^+^ signals were detected in the olfactory bulb, cortex, hippocampus, or cerebellum of any *Pcdh-*deficient mutants. Taken together, these findings suggest that the Pcdh proteins are exclusively essential for the survival of neuronal populations in the brainstem, including the midbrain, pons, and medulla, and in the spinal cord.

### Apoptosis of medullary interneurons is more severe in Δβγ and Δαβγ than in Δγ mutants

**Figure 10:**
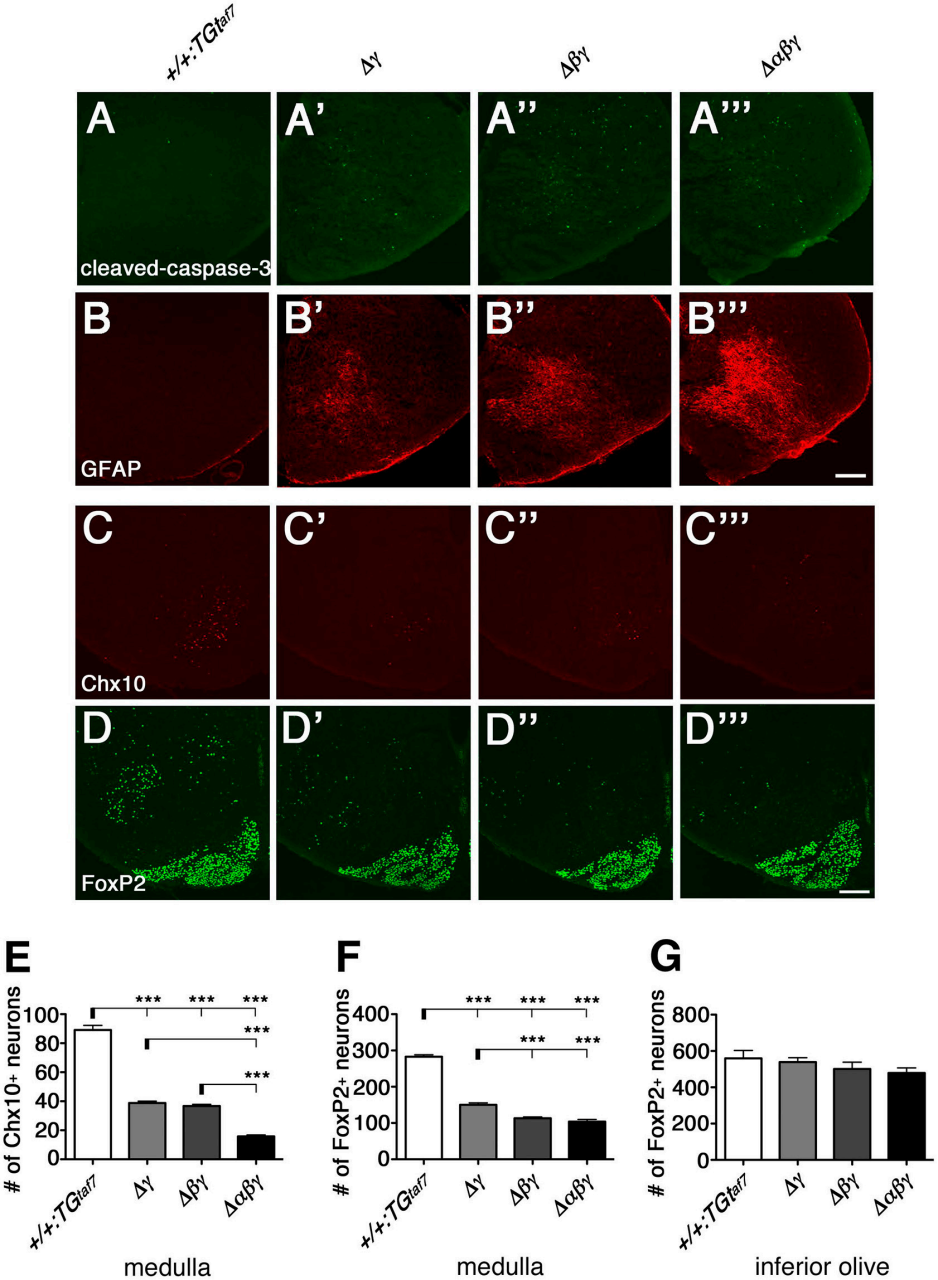
**Gliosis and Chx10^+^ and FoxP2^+^ interneuron subpopulations in the E18.5 medulla**. **(A–B”')** The E18.5 medulla, double-stained for cleaved-caspase-3 (green) and GFAP (red), showed massive astrogliosis in all three *Pcdh*γ-deletion mutants. The astrogliosis activation was markedly greater in the triple-deletion (Δ*αβγ*) mutant, which also had the highest level of neuronal death in the medulla at E16.5 **(A”')**. Thus, both the neuron-death phenotype and the severity of astrogliosis were correlated with the number of *Pcdh*-cluster deletions. **(C–D”')** Representative images of the E18.5 medulla stained for **(C–C”')** Chx10 and **(D–D”')** FoxP2. **(E,F)** Number of Chx10^+^ and FoxP2^+^ neurons in the medulla. Although excitatory Chx10^+^ and inhibitory FoxP2^+^ interneuron subpopulations were affected differently, the phenotypic severity of the double Δ*βγ* mutant was between that of the single Δγ and triple Δ*αβγ* mutants. **(G)** Note that the number of FoxP2^+^ neurons in the inferior olive was not altered, indicating that Pcdh clusters were dispensable for these neurons. *N* = 4–6 animals per genotype. Error bars are SEM. ^***^P < 0.001 by one-way ANOVA and Tukey's *post-hoc* test. Bars: 200 μm.

To investigate the neuronal loss in more detail, we analyzed coronal sections of the medulla of Δγ, Δ*βγ*, and Δ*αβγ* mutants. Because neurodegeneration typically causes astrogliosis, we stained for Glial fibrillary acidic protein (GFAP) to detect astrocyte activation, which accompanies neuronal-cell death in the E18.5 medulla of these mutants (Figures 10A,B). As expected, astrogliosis was most extensive in the Δ*αβγ* mutant, which also had the highest levels of neuronal-cell death in the medullary region at E16.5 (Figure 9A”').

We next quantified the medullary neuron-death phenotypes by counting Chx10^+^ excitatory (Figures 10C–C”') and FoxP2^+^ inhibitory (Figures 10D–D”') neurons in coronal sections of the E18.5 medulla. In single Δγ mutants, Chx10^+^ and FoxP2^+^ neurons were reduced to 43.5% and 53.1%, respectively, of the levels in control mice; the neuronal survival rates were 41.2% (Chx10^+^) and 40.0% (FoxP2^+^) in double Δ*βγ* mutants and 17.7% (Chx10^+^) and 36.8% (FoxP2^+^) in Δ*αβγ* mutants (Figures 10E,F). Notably, the survival rate of FoxP2^+^ neurons in the inferior olive was unaffected by the deletion of *Pcdh* clusters, suggesting that the *Pcdh* genes were dispensable for the survival of inferior olive neurons (Figure 10G, Katori et al., 2009).

### The neuron-death phenotype in the developing retina at E18.5

We extended our analysis of the neuron-death phenotype to the retina. While the five retinal layers are well developed by postnatal day (P) 18, the developing retina at E18.5 consists of only two layers: The neuroblastic layer (NBL) and the neural layer (NL). Although the neural circuits of the retina are immature, the retinal ganglion cells (RGCs) are already born at this time. RGCs are born by E12, starburst cells become postmitotic between E11 and E17, the internal plexiform layer (IPL) first appears at E17, and starburst amacrine cells have processes froming two discrete bands by P3 (Farah and Easter, 2005; Ford et al., 2012). On the other hand, neurogenesis and the migration of newborn interneurons and photoreceptors continue in the NBL after birth.

**Figure 11:**
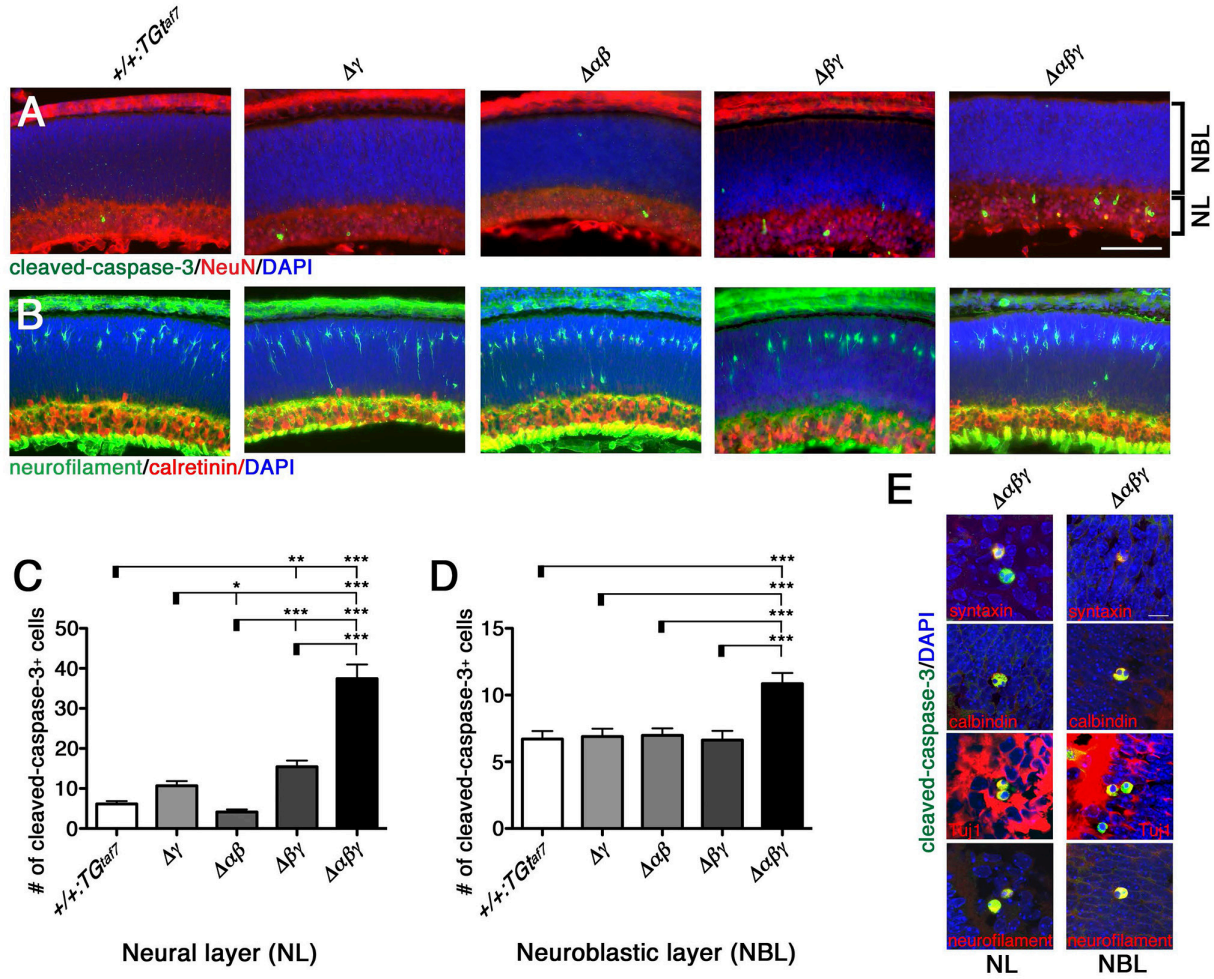
**Neuronal death phenotype in the E18.5 retina**. **(A)** E18.5 retinas were double-stained for cleaved-caspase-3 (green) and NeuN (red) and counterstained with DAPI (blue), or for **(B)** neurofilament (green; horizontal-cell marker) and calretinin (red; amacrine- and ganglion-cell marker). **(C,D)** Counts of cleaved-caspase-3^+^ retinal cells in five mutant animals. Counts in the NL differed significantly from the control in **(C)** for both the Δ*βγ* and Δ*αβγ* mutants; **(D)** NBL counts differed only in the Δ*αβγ* triple-deletion mutant. **(E)** Merged representative images of dying neurons double-stained for cleaved-caspase-3 (green) and the indicated proteins (red). *N* = 4–6 animals per genotype. Error bars represent SEM. ^***^P < 0.001, ^**^P < 0.01, and ^*^P < 0.05 by one-way ANOVA and Tukey's *post-hoc* test. Bars: 100 μm in **(A)**; 10 μm in **(E)**.

One study reported that Pcdhγ-null and hypomorphic mice at late embryonic stages (E17–18) have no obvious defects in retinal structure (Lefebvre et al., 2008). All of our mutant animals also appeared normal in the structure and thickness of the NBL and NL (Figure 11A), and in the distribution pattern of neurofilament^+^ (a horizontal-cell marker) and calretinin^+^ (an amacrine- and ganglion-cell marker) cells (Figure 11B). We next examined whether a neuron-death phenotype was present in the developing retina, as in the spinal cord and brainstem at the same embryonic stage (E18.5). Cleaved-caspase-3^+^ cells in the NL were significantly elevated in the Δ*βγ* and Δ*αβγ* lines compared to control animals (Figures 11A,C). In contrast, in the NBL, the cell-death phenotype was found only in the triple-mutant Δ*αβγ* animals (Figure 11D). We next calculated the ratio of NeuN+ neuronal cells to cleaved-caspase-3+ cells undergoing neuronal death. The average ratio (three animals per genotype) in the NL was 5.3% in +*/*+*:TG*^*taf7*^ and 12.2% in Δ*αβγ* animals; in the NBL, the ratios were 8.5% in +*/*+*:*TG^*taf7*^ and 16.0% in Δ*αβγ* animals. A previous study of Pcdhγ-deletion mutants reported that a phenotype of increased neuronal death began appearing in the retina in the postnatal stage (Lefebvre et al., 2008); although retinal defects were first noted after P0 in previous Pcdhγ mutants, retinal defects were already detectable in our Δ*βγ* and Δ*αβγ* mutants even at E18.5. Thus, although deleting both the *Pcdh*α and *Pcdh*β clusters enhanced the level and the time course of neuronal death in the single *Pcdh*γ mutant, the *Pcdh*γ cluster remained the main regulator for neuronal survival.

To identify the types of dying cells in the NL and NBL, we co-immunostained sections from Δ*αβγ* animals with cleaved-caspase-3 and cell-type-specific markers. We stained for the following markers: Neurofilament and calbindin for horizontal cells; syntaxin, calbindin, and ChAT for amacrine cells; Chx10 for bipolar cells; and Tuj1 and calbindin for ganglion cells (Figure 11E). We found cleaved-caspase-3^+^ dying neurons that were also positive for syntaxin, calbindin, Tuj1, and neurofilament. These were putative amacrine or ganglion cells in the NL and putative horizontal cells in the NBL. In fact, differentiation, layer formation, and synaptic maturation occur earlier in these two cell types than in other cell types (Young, 1985), suggesting that the three Pcdh clusters may cooperatively contribute to these early differentiation mechanisms through their molecular specificity and the diversity of the Pcdhα, Pcdhβ, and Pcdhγ proteins.

## Discussion

### Pcdhβ-cluster functions

In this study, we found that Pcdhβ isoforms are important for olfactory-axon targeting and interneuron survival. They have homophilic binding activity and form *cis*-hetero dimers with other clustered Pcdh proteins. However, Pcdhβ proteins do not have a common cytoplasmic domain, unlike the Pcdhα and Pcdhγ proteins. Interestingly, Pcdhβ-deletion mutants added to Pcdhα or Pcdhγ mutants resulted in additional abnormalities in olfactory-axon coalescence, suggesting that Pcdhβ isoforms cooperate with Pcdhα and Pcdhγ isoforms as *cis*-hetero dimers. Also, double-deletion mutants with Pcdhγ had additional neurodegenerative abnormalities in Lamina I–III and VI–VIII neurons, in spinal-cord Chx10^+^ and FoxP2^+^ neurons, and in FoxP2^+^ neurons in the medulla, and also had high numbers of excitatory VGluT1^+^ puncta surrounding the MNs. These additional phenotypes suggested that Pcdhα and Pcdhβ isoforms function cooperatively in the medulla and the spinal cord. All of the Pcdhβ isoforms have stochastic and combinatorial expression in individual neurons, suggesting that the stochastic and combinatorial expression of diverse clustered Pcdhs regulates olfactory-axon targeting and interneuron survival. Here, we presented multiple findings suggesting that the combinatorial diversification of *cis*-hetero dimers among the clustered Pcdhs in the brain provides both cooperative functions and diversity in individual neurons. The phenotypes of the Pcdh-deletion mutants are summarized in Table 1.

**Table 1:** **Phenotypes of *Pcdh*-deletion mutants**.

**Phenotypic severity**	**Deletion mutant lines**					
	**Δα**	**Δβ**	**Δγ**	**Δαβ**	**Δβγ**	**Δαβγ**
Olfactory axon	+	+	+	++	++	++
Ia afferent axon	−	−	+	−	+	+
Neuronal death (Spinal cord)	+	+	++	+	+++	+++
Neuronal death (Medulla)	NE	NE	+	NE	++	+++
Neuronal death (Retina)	NE	NE	−	−	+	++

*NE; not examined*.

### Neuronal diversity

The molecular diversity of clustered Pcdhs has led to the proposal that they provide neuronal diversity for neural wiring or for self-recognition (Lefebvre et al., 2012; Yagi, 2012, 2014; Rubinstein et al., 2015). The clustered *Pcdh* genes are attractive candidates for determining neuronal diversity at the molecular level, because they differentially express distinct subsets in each neuron. However, their biological significance in neurons remains largely unclear. Here we established various combinations of deletion mutants for the *Pcdh*α*, Pcdh*β, and *Pcdh*γ clusters, and obtained the first evidence that the *Pcdh*β cluster also regulates neuronal survival. All of the *Pcdh*β-cluster isoforms are known to have random combinatorial expression in single neurons and to provide neuronal diversity in the brain (Hirano et al., 2012; Yagi, 2012, 2014). These features indicate that the *Pcdh*β isoforms have a separate role that requires their diversity and is important for neuronal survival during embryonic development. One study showed that genetically blocking apoptosis with *Bax* mutants rescued the neonatal lethality of triple C-type isoform KO mutants, but not for total *Pcdh*γ-KO mutants, indicating that the remaining 19 A- and B-type *Pcdh*γ genes, which are randomly expressed in single neurons, have a separate role that is essential for postnatal development (Chen et al., 2012). These studies suggested that *Pcdh* isoforms that are randomly regulated at a single-neuron level are essential for interneuron survival in the brainstem and spinal cord. On the other hand, constitutively expressed triple γ*C* isoforms are also important for interneuron survival. The triple γC isoform proteins can combine to form heterodimers with the Pcdhβ, PcdhγA, and PcdhγB isoforms (Thu et al., 2014); therefore, a wide range of highly diverse oligomers is expressed in each neuron. Thus, the diversification of Pcdh oligomers may have important roles in interneuron survival, and probably in neural network wiring.

### Distinct function of Pcdhγ in Ia afferent arborization

Interestingly, even in the triple *Pcdh*α-, *Pcdh*β-, and *Pcdh*γ-deletion mutants, the defects in Ia primary afferents were similar to those in the single *Pcdh*γ-deletion mutant. The degree of abnormality of the Ia primary afferents is similar in triple C-type isoform KO mutants and in *Pcdh*γ-KO mutants. These data suggest that the normal axon targeting of Ia afferents does not require the neuronal individuality resulting from Pcdh diversity, and that Ia afferent arborization is mainly regulated by only the triple C-type Pcdhγ isoforms γC3, γC4, and γC5. Pcdh diversity is not required for these Ia afferent arborizations. Therefore, the C-type isoforms, which are constitutively expressed in neurons, possess distinct functions from those of the stochastically expressed Pcdhα, Pcdhβ, and Pcdhγ isoforms. These experiments focusing to molecular diversity and specificity might be done in the future to determine whether cell surface diversity might be important for axon coalescence mechanism.

### Distinct and cooperative functions in olfactory axon projections

The *Pcdh*α cluster and the common cytoplasmic tails among the Pcdhα isoforms are essential for normal glomerular formation by olfactory-axon coalescence (Hasegawa et al., 2008). Interestingly, the glomerular formation is normal in *Pcdh*α*1*-expressing mice, mutants with a deletion of the α*2* to α*C2* genes (Hasegawa et al., 2012). These results indicate that normal glomerular formation and axon coalescence do not require diversity within the Pcdhα isoforms. However, our current data clearly showed that not only Pcdhα, but also distinct functions of each Pcdhβ and Pcdhγ protein (at least one isoform in each cluster) are required for normal olfactory-neuronal circuits. It is reported that Pcdhγ is important for the proper maturation of postnatally generated olfactory bulb granule cells (Ledderose et al., 2013). In the future we need to determine whether the amount of cell-surface diversity, based on Pcdh-isoform *cis*-hetero dimers, or cytoplasmic signals of Pcdhα and Pcdhγ plays an important role in the axon coalescence of olfactory sensory neurons.

### Distinct and cooperative functions for spinal-cord interneuron survival

When analyzing the neuron-death phenotype in the spinal cord, we found that the Pcdhγ cluster was the primary agent in the survival of spinal interneurons. However, we observed neuronal death phenotype even single *Pcdh*α- or *Pcdh*β-deficient mutant embryos. Therefore, the *Pcdh*α*, Pcdh*β, and *Pcdh*γ clusters are all required, to various degrees, for interneuron survival in the spinal cord during late embryonic development. Because more than 70% of the Chx10^+^ and FoxP2^+^ interneurons escaped death in the single *Pcdh*α- or *Pcdh*β-deficient mutant, these mice exhibited normal gross phenotypes. These results suggested that the molecular diversity of the clustered Pcdhs is important in forming functional interneuronal networks in the brainstem and spinal cord.

A similar neuronal apoptosis phenotype is reported in the developing zebrafish. Truncating Pcdh1α proteins by antisense morpholinos causes apoptosis in neurons throughout the developing brain and spinal cord (Emond and Jontes, 2008). The structures of the clustered *Pcdh* genes differ between mammals and zebrafish, which have two *Pcdh*α gene clusters (10 *Pcdh1*α and 35 *Pcdh2*α); nevertheless, this finding suggests that the clustered *Pcdh* genes have evolutionarily conserved functions for regulating neuronal survival.

### Neuronal death in the retina

In contrast to spinal cord and brainstem, Pcdhγ was not required in the prenatal retina, which exhibited neuronal survival in the single Pcdhγ-deletion mutant. In the E18.5 retina, the neuron-death phenotype was found in double Pcdhβ- and Pcdhγ-deficient mutants and triple Pcdhα-, Pcdhβ-, and Pcdhγ-deficient mutants. Our present study showed that amacrine, ganglion, and horizontal cells were especially sensitive to the loss of Pcdh proteins. Interestingly, self-avoidance is also reported in the horizontal, bipolar, amacrine, and ganglion cells of the retina, as well as in cerebellar Purkinje cells (Montague and Friedlander, 1991; Wässle et al., 2009; Lefebvre et al., 2012; Matsuoka et al., 2012). In addition, analogous to the spinal cord and medulla, these retinal neurons except for ganglion cells are interneurons that are born and differentiate relatively early in retinal development. Thus, most differences in how many neurons would be dependent on the maturity of the cells and developmental timing, rather than actual different requirements for *Pcdh* clusters in survival of some neurons and not others.

In this study, we show the distinct and cooperative function of clustered protocahderins during brain development. However, we need to make a lot progress toward understanding what the clustered protocadherins do, or to what extent the hetero-oligomerization influences function *in vivo*. Here deletion mutants will be useful tools to address their questions.

## Author contributions

SH and TY designed the research and wrote the manuscript. SH and MK performed the immunohistochemistry and analyzed the data. M. Hagihara performed the immunoprecipitation. HN performed the electrophysiological analysis. MW produced anti-Pcdhα and anti-Pcdhγ antibodies. T. Hirabayashi performed the RT-PCR and immunoblot analyses. T. Hirayama performed the quantitative RT-PCR analysis. KH, RK, AO, MS, M. Hirabayashi, and T. Hirayama contributed to generating the mutant mice.

### Conflict of interest statement

The authors declare that the research was conducted in the absence of any commercial or financial relationships that could be construed as a potential conflict of interest.
