# Supplementary material for: Distinct and Cooperative Functions for the Protocadherin-α, -β and -γ Clusters in Neuronal Survival and Axon Targeting
Source: Front Mol Neurosci. 2016 Dec 23;9:155. doi: 10.3389/fnmol.2016.00155 (PMC5179546; doi:10.3389/fnmol.2016.00155)
Supplement: Supplementary file 1 [file Table1.DOCX]

**Supplementary Table S1. List of primer sequences**

**<For genotyping Pcdh-cluster deletion-mutant mice>**

P1: 5’- GCTTATTTGCTAGAACGTCC - 3’

P2: 5' - CGGAGCCTGGAAAACAGCAT - 3'

P3: 5’- GCTTATTTGCTAGAACGTCC - 3’

P4: 5' - GCTCCTGATTGAATTTGCC - 3'

P5: 5’- CCGCTTCCTCGTGCTTTAC- 3’

P6: 5' - TTTGGTGCATCCATTTGGAGTGTGG - 3'

P7: 5’- TGATGTGGGTCTGGTTTCC - 3’

P8: 5’- GCCTTTATTGACTATAGTGCACC - 3’

P9: 5'- TTGTGAGTGCTCCATAGCCTC - 3'

P10: 5' - TTTGGTGCATCCATTTGGAGTGTGG - 3'

P11: 5' - AGCCTAAGGGTCACAATGGAAGC - 3'

P12: 5' - CCAAGGCGACAGAGAAGGAAG - 3'

T1: 5'-GCGCGCCAAAGCTTGCATGC-3'

T2: 5'-CTCTCCCTATAGTGAGC-3'

**<For generating mutant mice with *loxP*-site insertions>**

**Construction of targeting vectors**

***α1MV* targeting vector**

a1MVA-F: 5' - CGGTCGACGTCATGTACAAGTTCTATGCC - 3'

a1MVA-R: 5' - CGGTCGACGATCATACTTTGCACCTTCATG - 3'

a1MVB-F: 5' - CGGGATCCTTGGTGTGACAGCGATACGG - 3'

a1MVB-R: 5’- CCATCGATCAGATCCTCTTCAGATGAGTTTCTGCTCAATCTGGCCGCTCCCTGCC - 3'

a1MVC-F: 5' - CCATCGATCGATCTCTCGAGATGGTGAGCAAGGGCGAGG - 3'

a1MVC-R: 5' - CCTGCAGGCTTGTACAGCTCGTCCATGC - 3'

a1MVD-F: 5' - CCATCGATCCTGCAGGCCAGATTCATTACTCTGTCCC - 3'

a1MVD-R: 5' - CGAGCTCGCGTCGACGTTCGTCTGTTGTCTCTACC - 3'

a1MVE-F: 5' - CCCAAGCTTAATGCGGCCGCCAACTAGCTCTGTAGACCAGG - 3'

a1MVE-R: 5' - ACGCGTCGACGCATTTATCATTCTGTCATGAGGG - 3'

a1MVF-F : 5' - ACGCGTCGACAGGTCGCTTGACTCAAGGTTTCC - 3'

a1MVF- R: 5' - CTAGCTAGCGACCTGGATTTCATAGGATGTCC - 3'

***αCR-loxP* targeting vector**

CP3/RT5C-F: 5' - AGCATGCGTCTTGTTCCCAGGTAAGTTTCC - 3'

CP3/RT5C-R: 5' - AGCTAGCGATTTCTGAGGGCTCATGTCA - 3'

CP3/RT3C-F: 5' - AGCTAGCCTGCATTAACTGAGAGGGAAGAC - 3'

CP3/RT3C-R: 5' - AAAGCTTCACTGACTTCTAGAACCAAACCC - 3'

CP3/BT5-F2 : 5' - AGGTACCGGTTACTTTGAAAGGGACTCCTAC - 3'

CP3/BT5-R : 5' - AAAGCTTCTCCATGAAAGGAAACGTGTGC - 3'

CP3/BT3-F: 5' - AGGATCCCTTGGCTGCTGATGAGCAG - 3'

CP3/BT3-R: 5' - AGAGCTCGGCTAGAGAGCTCCATGC - 3'

***β1-loxP* targeting vector**

b1loxA-F: 5' -GGTACCGGCTGTTTCTGTCCATTGG - 3'

b1loxA-R: 5' -AAGCTTCACCCCCTAAATTGTTTGC - 3'

b1loxB-F: 5' - GCGGCCGCGAGCTCACACAGAAACAGAGCAATG - 3'

b1loxB-R: 5' - CCGCGGCGAAGCATAAAGGAAACAC - 3'

b1loxC-F: 5' - AACGTTCTCGAGGGTATAGCCAGCAGTTCTG - 3'

b1loxC-R: 5' - GTCGACATGCCACCATAATGTCAAG - 3'

b1loxD-F: 5' - GTCGACGTTGATCTTTATTGTAAGC - 3'

b1loxD-R: 5' - GCTAGCATGTATATTTCATTTTAGC - 3'

***β22-loxP* targeting vector**

b22loxA-F: 5'- GCGGCCGCATCATCTCATGGAAACCAGACCCAC - 3',

b22loxA-R: 5'- GCGGCCGCGTCGACGGCATTCTGTACAACCAGAAACTACCC - 3'

b22loxB-F: 5'- ACGCGTCGACGCTCTGAGGGACAGCAGAGAGG - 3'

b22loxB-R: 5'-CTAGCTAGCCAGCTTCTGTCCACTGGTTGG - 3'

b22loxC-F: 5'-CCATCGATGGTGCTTTCATATAAGCTTTTCC - 3',

b22loxC-R: 5'-ACGCGTCGACCAACAGATTGCAGCATGGTTCC - 3'

b22loxD-F: 5'-GCGATATCACATCAGGACACTTCACGCTCC - 3'

b22loxD-R: 5'-CCATCGATCTCGAGTGAGGCTACTGAAGTCATAGCC - 3'

**<Isolation of probes for Southern hybridization analysis>**

**Probe A**

F: 5' - GAAATGAAATTTGATAGATGG - 3'

R: 5' - AATACAACACATTTCCAACC - 3'

**Probe B**

F: 5'- ACAGAGAGGACATTCCTG - 3'

R: 5'- ATGACCAATTCAGGCACG - 3'

**Probe C**

F: 5'- CTTAATCTGCCATGTCACAG - 3'

R: 5'- GATGGAAAGGTGACGCAA - 3'

**Probe D**

F: 5' - TTTATTTTGTCTGAGTTTG - 3'

R: 5'- ATTGTGTTTCTCTGGTTAC - 3'

**Probe E**

F: 5'- ATAAACAACTTCAGCAAAG - 3'

R: 5'- TACCACCATTTCTCTGTAG -3'

**Probe F**

F: 5' - GCCTGGATTAGTGAACTATACCTG - 3'

R: 5' - CCTGTAAGTACTACCCATTGCTGG - 3'

## <For RT-PCR analysis>

**Pcdh-αCR**

F: 5' - CCGGCAGCCCAACCCTG - 3'

R: 5' - GAGATGATTGCAGGAGATCCTGGG - 3'

**Pcdhβ5**

F: 5' - GGTTCAGGATACGGGAAATCTTC - 3'

R: 5' - CAGCACTGTAAGAGATATAAGTAGG - 3'

**Pcdhβ8**

F: 5' - TGCATTAACTGTGCAAAGTACAGCG - 3'

R: 5' - GAGACTGATGGGGAGGAAG - 3'

**Pcdhβ16**

F: 5' - GTTCTGGGATGGTTTGGAAATGTAC - 3'

R: 5' - GACCTCGTTGTGTTTGAGCATTG - 3'

**Pcdhβ22**

F: 5' - AACTATGGTAGGCAACCAGATGATC - 3'

R: 5' - GAATACAGAGAGCGAAATGTGACG - 3'

**Taf7**

F: 5' - ATTCCAGCTCTTCCTGCAAA - 3'

R: 5' - ATGAAAGGCAAGCTCCAAGA - 3'

**PcdhγCR**

F: 5' - CCGCCCAACACTGACTGGC - 3'

R: 5' - CATCTCGTTTGCCAGCGGCATTG - 3'

**<For quantitative RT-PCR analysis>**

**Taf7**

F: 5' - AATCCGACGAGCAACACCAAG- 3'

R: 5' - GGTTTCTTGGAGCTTGCCTTTC- 3'

**Gapdh**

F: 5' - AATGTGTCCGTCGTGGATCT- 3'

R: 5' - GTTGAAGTCGCAGGAGACAA- 3'
